# Using machine learning and qualitative interviews to design a five-question survey module for women’s agency

**DOI:** 10.1016/j.worlddev.2022.106076

**Published:** 2023-01

**Authors:** Seema Jayachandran, Monica Biradavolu, Jan Cooper

**Affiliations:** a Princeton University, United States; b QualAnalytics, United States; c Harvard University, United States

**Keywords:** Women’s empowerment, survey design, feature selection, psychometrics

## Abstract

Open-ended interview questions elicit rich information about people’s lives, but in large-scale surveys, social scientists often need to measure complex concepts using only a few close-ended questions. We propose a new method to design a short survey measure for such cases by combining mixed-methods data collection and machine learning. We identify the best survey questions based on how well they predict a benchmark measure of the concept derived from qualitative interviews. We apply the method to create a survey module and index for women’s agency. We measure agency for 209 married women in Haryana, India, first, through a semi-structured interview and, second, through a large set of close-ended questions. We use qualitative coding methods to score each woman’s agency based on the interview, which we use as a benchmark measure of agency. To determine the close-ended questions most predictive of the benchmark, we apply statistical algorithms that build on LASSO and random forest but constrain how many variables are selected for the model (five in our case). The resulting five-question index is as strongly correlated with the coded qualitative interview as is an index that uses all of the candidate questions. This approach of selecting survey questions based on their statistical correspondence to coded qualitative interviews could be used to design short survey modules for many other latent constructs.

## Highlights

• Surveys often aim to measure complex concepts with a few close-ended questions. We introduce a new method for selecting questions.
• The method identifies the survey questions that best correspond to answers given in a qualitative interview about the concept.
• We field many survey questions and use machine learning (ML) tools to find the questions that best predict the coded qualitative data.
• We apply the method to create a five-question survey module to measure women’s agency in north India.
• The questions chosen are similar across three ML algorithms, and the resulting survey measure of women’s agency performs well in diagnostics.

## 1 Introduction

Researchers often want to use a few close-ended survey questions to measure a psychological construct, or postulated attribute (Cronbach & Meehl, 1955). An example of such an attribute is agency, or a person’s ability to make and act on the choices for her life. In research related to gender, social scientists might want to test whether an intervention increased women’s agency (i.e., use the measure as an outcome variable) or investigate whether women with more agency enjoy larger benefits from an intervention (i.e., use it for subgroup analysis). An accurate and precise measure of agency is important for these purposes.

Agency is not directly observable, and it is multi-faceted: It encompasses the many domains of a person’s life including reproductive health, employment, and household finances, and it is defined as having both instrumental and intrinsic value to a person.^1^ The complexity of agency makes it a challenge to measure quantitatively. While this complexity suggests the need for a long survey module, researchers often seek a short module, particularly if agency is a secondary focus of their study. Administering a longer module would require more money and more of respondents’ time.

In this study, we develop a new method to select a few survey questions to measure a latent construct. The method delivers a set of survey questions of a desired length and an index (i.e., a way of aggregating the responses to the questions into a scalar). We apply the method to create a five-question module for women’s agency using data collected from married women in rural Haryana, India.

How well a psychometric captures the concept it is trying to measure is called its validity (DeVon et al., 2007, Jose et al., 2017). Our method draws on the idea of criterion validity, or the correspondence between a proposed measure and a “gold standard” measure of the same construct.^2^ We conduct in-depth, semi-structured interviews with women, which allow for probing questions and elicit rich responses about their agency. We then use qualitative coding methods to construct a scalar measure of agency based on the qualitative interview, which we use as a benchmark measure of agency. Finally, we select the best five questions to use, from among a large set of contenders, based on their statistical correspondence with the benchmark measure.^3^ To do so, we apply feature selection algorithms that build on standard supervised machine learning techniques, adding a constraint on the number of survey questions that are selected.

We refer to this new approach to survey module design as MASI, for MAchine learning and Semi-structured Interviews.^4^ Many complex concepts in the social sciences are best investigated by asking open-ended questions, yet there is practical need for close-ended measures of them. One could apply MASI to create survey modules for other constructs, such as financial insecurity or cultural assimilation.

If a richer measure based on semi-structured interviews exists, then why not always use it? Because the measure is time- and skill-intensive, and thus expensive, to collect, making it infeasible for large-*N* studies. We collect the richer measure for a relatively small sample to serve as a benchmark in a one-time exercise. The short survey module developed using this small sample can then be incorporated into large-*N* surveys, with some confidence that it provides an accurate measure of the construct, despite its brevity.

To implement this approach, we collected data on women’s agency in multiple ways. First, trained qualitative researchers conducted semi-structured interviews. These interviews provide nuanced data but require highly skilled staff to conduct and code them. Second, we collected another candidate benchmark measure of agency using a lab game. In the game, which we adopt from Almås, Armand, Attanasio, and Carneiro (2018), each woman makes a real-stakes choice between money for herself or her husband. This lab game adds logistical complexity and costs to the fieldwork, but observed behavior might be less subject to social desirability bias than survey responses. We pursued these two quite different benchmarks out of recognition that researchers likely differ in which they prefer, according to their methodological taste. We conduct the lab game among 443 women and choose a subsample of 209 of them for the semi-structured interviews. The lab game, however, was ineffective in measuring women’s agency in our sample. We therefore discuss the data collection using the lab game, but focus on the qualitative interviews as the benchmark measure in our statistical analysis to derive a short survey module.

The third way we measure women’s agency is through close-ended survey questions. We ask a long list of questions, drawing on existing survey instruments. Our objective at the data collection stage was to be comprehensive and agnostic about which were the best questions, and then to later use a data-driven approach to select the best ones. There is nothing special about five questions, but this length seems appropriate for survey designers seeking a short module on agency.

The goal of our statistical analysis is to identify the best close-ended questions to field from among many candidates. The algorithms we use build on standard supervised machine learning techniques, adding a constraint on the number of survey questions that are selected. This type of problem is referred to as feature selection. We apply three feature selection algorithms. Our preferred algorithm is LASSO stability selection, in which the top questions are those selected most frequently when LASSO is repeatedly run on subsamples of the data (Meinshausen & Bühlmann, 2010). This method has previously been used by Kshirsagar, Wieczorek, Ramanathan, and Wells, (2017) to choose a small set of survey questions for a proxy-means test of household poverty, for example.^5^ In our view, this algorithm strikes a good balance between transparency of the predictive model, ease of implementation, and avoidance of over-fitting the data. The second algorithm is a more complex procedure using random forest that has more flexibility to fit non-linear relationships in the data (Genuer, Poggi, & Tuleau-Malot, 2010). The third algorithm, backward sequential selection, is more prone to over-fitting but is the simplest one (Liu & Motoda, 1998). It uses only standard linear regressions. We start with the full set of survey questions and iteratively remove the question that leads to the smallest decrease in the set’s explanatory power, stopping when the desired number of questions remain.

Turning to our results, when we use the qualitative interviews as the benchmark measure, all three of the statistical algorithms produce an index of women’s agency that is quite strongly correlated with the interview score. There is considerable overlap in the top questions selected by each algorithm. In addition, the five-question indices are considerably more correlated with the benchmark than if we had chosen the subset of questions randomly. They also perform better than indices constructed from all 63 candidate questions, either their first principal component or a standardized index that averages them. Interestingly, the algorithm-selected questions are quite specific ones about decision-making in particular situations, rather than questions that ask women about their power in general.

In the lab game, the premise is that a woman with less agency will more often choose money for herself because she would not have a say in how money given to her husband is spent. We do see this behavior, but we also see an opposing force: some women with very low agency never want money for themselves because they view money as men’s domain or are fearful of their husband finding out and becoming angry. Even when we take into account this bimodal behavior – women with low agency either have very high or very low demand to receive money themselves – the survey index obtained when we apply the statistical techniques is only weakly correlated with the lab game behavior. We conclude that only the semi-structured interviews can be considered a good benchmark measure of agency in our setting. Another advantage of the qualitative interviews is that they cover many domains of agency, not just financial agency.

The primary contribution of our study is methodological: We introduce a new mixed-methods way to develop a survey measure. Using qualitative methods in the design of measurement scales is not new (Onwuegbuzie et al., 2010, Zhou, 2019). For example, Creswell and Clark, 2017 describe a process of using qualitative methods to define a construct and then quantitative methods to assess the scale once it is developed.^6^ In addition, machine learning techniques have been used in the development of survey instruments, for example to pare down full-length scales to short-form versions (Gonzalez, 2020). What is new is to select quantitative questions algorithmically by using a qualitative measure as the benchmark, or as the “labels” supplied to the machine learning algorithms.

A second contribution of our study is the new short survey module and index for women’s agency that we develop. Our study thus adds to the literature proposing measures of women’s agency or empowerment, which we review in the next section. We created a module optimized for use in north India. One direction for future research is to replicate the study elsewhere to create short modules appropriate for other contexts and to assess the extent to which the same questions are or are not selected elsewhere. One could also apply our method to design a “universal” module based on how robustly it predicts qualitative interview scores across multiple contexts.

## 2 Related literature on women’s agency

### 2.1 The concept of agency

Agency is one aspect of women’s empowerment. Empowerment as defined by Kabeer, 1999 encompasses resources, agency, and achievement and refers to the process of acquiring the ability to make choices. Contemporary notions of empowerment often build on Amartya Sen’s capabilities approach, as elaborated by Nussbaum, 1999, who highlights that dignity and the freedom to actively determine one’s life are central to human beings.

Agency specifically refers to the ability to make decisions and act on one’s goals. It is often defined in a way that captures both an intrinsic characteristic and something with external, instrumental value. To do this, many definitions of agency reflect both an internal feeling of agency (sometimes defined as the ability to set goals, where the setting of goals is a reflection of the intrinsic sense of agency) and the external actions of pursuing goals, which is the instrumental aspect of agency (Donald, Koolwal, Annan, Falb, & Goldstein, 2020).

Scholars have also highlighted that the conceptualization of women’s agency depends on the context, for example differing in more coercive settings. Individual actions must be viewed within social, economic, and cultural contexts, and there are multiplicities and hidden forms of women’s agency (Campbell & Mannell, 2016).

### 2.2 Measurement of women’s agency

There is an array of research on how to measure women’s empowerment and agency. Donald et al. (2020) and Laszlo, Grantham, Oskay, and Zhang (2020) provide excellent overviews of this literature.

Recent proposed measurement tools include the Women’s Empowerment in Agriculture Index (WEAI) (Alkire et al., 2013) and PRO-WEAI (Malapit et al., 2019). WEAI is a set of survey questions that measures empowerment, agency, and inclusion in the agricultural sector (Alkire et al., 2013). It aggregates an individual’s empowerment across five domains and also measures women’s status relative to men in the household. The index was designed based on analysis of household survey data collected in Guatemala, Uganda, and Bangladesh, and it has been applied in several other contexts subsequently. PRO-WEAI adapts the WEAI to measure empowerment brought about by agriculture projects (Malapit et al., 2019). It includes further indicators that are most likely to change over the course of a project’s duration. This adaptation of the WEAI was informed by qualitative data from key informants and project participants.

Another measure is the Survey-based Women’s Empowerment Index (SWPER), which was developed by analyzing responses to Demographic and Health Survey questions among partnered women in 34 African countries (Ewerling et al., 2017). SWPER includes 15 questions that represent three dimensions of empowerment: attitudes toward violence, social independence, and decision making. SWPER was adapted into a 14-question version designed to be applicable in all low- and middle-income countries (Ewerling et al., 2020). Another recent contribution is by Maiorano, Shrimankar, Thapar-Björkert, and Blomkvist, 2021, who introduce a choices-values-norms framework for measuring agency. Specifically on India, Kishor and Gupta, 2004 adapt WEAI for nutrition, while Richardson, Schmitz, Harper, and Nandi, 2019 develop an index of National Family Health Survey questions using confirmatory factor analysis.

A different strand of the literature assesses current practices for measuring women’s agency. Donald et al. (2020) and Laszlo et al. (2020) highlight conceptual challenges and provide frameworks to guide measurement. Other efforts have documented which measures have been tested at scale across settings and which require further testing, as well as current gaps where the creation of new measures is needed (Center on Gender Health & Equity, 2020). Also, domain-specific literature syntheses reveal that measurement efforts have often concentrated on certain dimensions of agency, with the measurement of other dimensions remaining under-developed (Bhan et al., 2020). Other work tests the sensitivity of findings to how agency is measured. For example, Peterman, Schwab, Roy, Hidrobo, and Gilligan (2021) investigate how robust results are to different ways of constructing agency indicators from commonly-used survey questions. They conclude that current practices are often insufficient to capture women’s decision-making and call for further measurement innovation.

## 3 Description of study site and sample

### 3.1 Selection of study site and sample villages

We selected Kurukshetra district in the Indian state of Haryana as the study site based on several considerations. We chose north India because of our knowledge of the context and because women’s agency is an important topic of study there. To match our team’s language skills, we restricted attention to Hindi-speaking areas. Within this narrowed set of possible sites, we chose Kurukshetra for practical reasons. First, we could draw on a pool of female surveyors who had worked on earlier studies conducted by J-PAL South Asia, the research organization through which our fieldwork was conducted. Second, the main town was large enough that we could recruit two lead research assistants from New Delhi who would be willing to be based there for several months. Third, Kurukshetra was within a few hours of New Delhi by car or train, which facilitated site visits by the principal investigators.

We focused on the rural population and worked backwards from our target sample size of 210 semi-structured interviews to determine how many villages within Kurukshetra to include in our sample. We were able to recruit two qualitative interviewers, and about 100 interviews each was the most they could conduct within the three months we had planned for the data collection. We wanted to complete data collection in each village within two or three days so that there would not be discussion among women about our study that might prime their answers. We expected each interviewer to conduct two to three interviews per day, which implied that our team should conduct about 10 qualitative interviews per village. We thus included 21 villages in our sample in order to complete roughly 210 interviews.

We had a separate, larger team of surveyors that conducted the quantitative surveys and lab game. The quantitative team spent about the same number of days in each village, collecting data from twice as many women. The final sample size for that team was 443 women, of whom the 209 semi-structured interviewees are a subset.

**Fig. 1:**
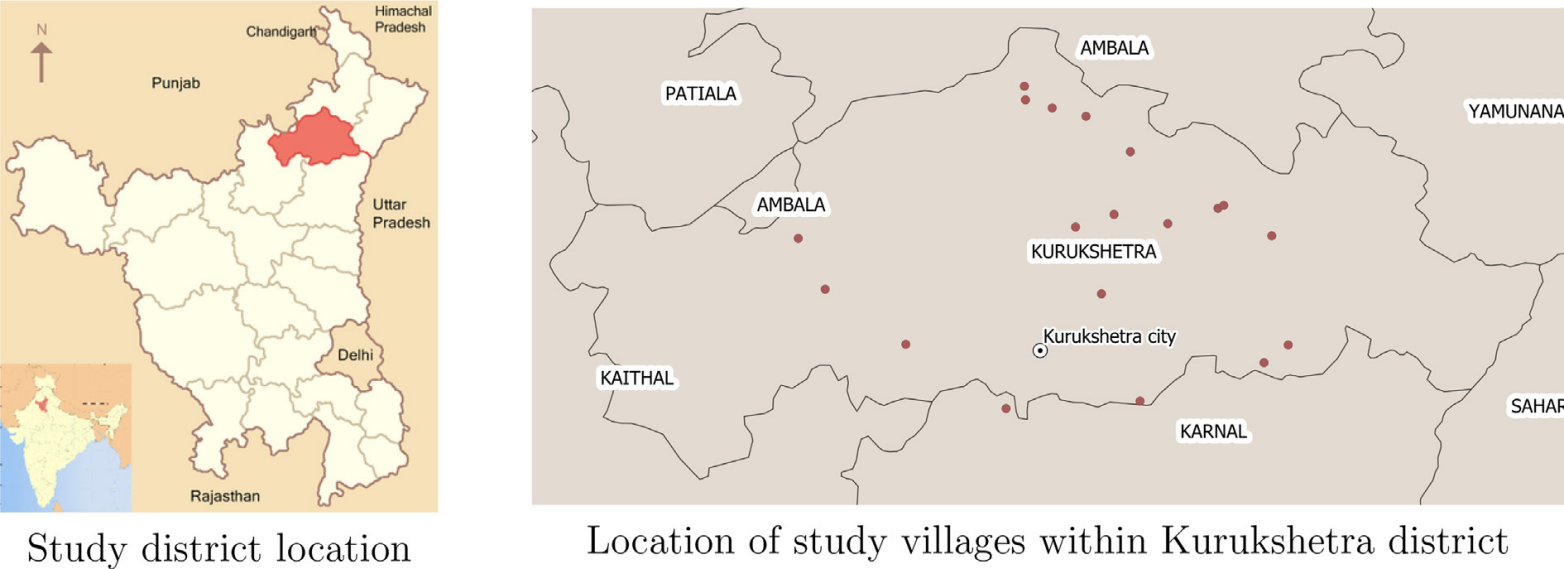
Study location.

We chose a random sample of villages for the study that were representative of Kurukshetra, with the selection stratified by village population, distance from the district headquarters, and the ratio of male to female literacy.^7^ We created a randomly ordered list of potential sample villages. We then visited the first 21 villages to obtain a roster of households with young children from the village ASHA, or Accredited Social Health Worker. We used these rosters to choose households for the sample. In the few cases where we could not obtain a roster from the ASHA, we replaced the village with the next village from its stratum on our list. Fig. 1 shows the location of Kurukshetra district within India and the location of the 21 study villages.

### 3.2 Selection of study participants and descriptive statistics

We used the ASHA lists to choose a preliminary random sample of eligible women in each village. Our eligibility criterion was that a participant was a married woman with a child under the age of 10; we wanted the sample to be homogeneous in this way so that we could ask everyone similar questions, for example about their relationships with their husbands and about decisions over children’s health. The ASHA data included a household roster but not relationships among household members, so we chose households with a child under age 10 and a woman at least 15 years older than that child, who was feasibly the child’s mother. We aimed to enroll 20 women per village (with no more than one enrolled woman per household) in the study, and we randomly chose 50% of them for the semi-structured interview.

We collected the data between February and May 2019. We varied whether the qualitative or quantitative data collection came first. The quantitative team started fieldwork in a random half of villages, and the qualitative team started in the other half; halfway through the data collection, they switched villages. (We do not find significant differences in measured agency, either qualitative or quantitative, based on the order of data collection.)^8^

The first step when the first team visited a household was to verify the woman’s eligibility for the study, which also required that she speak Hindi.^9^ We then explained the study and obtained informed consent.

Table 1 reports summary statistics for the sample, based on data collected in the quantitative survey. The women are on average 30 years old with a youngest child who is five years old. Women are, on average, 3 years younger than their husbands. The average years of schooling is 10. Most of the sample is Hindu; Sikhism is the second most common religion. About a third of the sample belongs to a scheduled caste or scheduled tribe, and about half belong to an ‘other backward caste.’ Less than a fifth of women are employed, consistent with the low India-wide female employment rate.

**Table 1:** Descriptive statistics for the sample.

Variable	Full sample	Sample with qual. interview
Number of respondents	443	209
Age	29.720	29.512
	[4.953]	[4.778]
Age at marriage	20.377	20.316
	[2.584]	[2.708]
Husband-wife age gap	2.946	2.914
	[2.821]	[2.702]
Age of youngest child	4.989	5.019
	[2.765]	[2.792]
Can read and write	0.986	0.986
	[0.116]	[0.119]
Years of education	9.916	10.024
	[3.258]	[3.175]
Husband-wife education gap	0.853	0.660
	[3.070]	[3.313]
Employed	0.165	0.182
	[0.371]	[0.387]
Hindu	0.840	0.837
	[0.367]	[0.370]
Sikh	0.151	0.144
	[0.359]	[0.351]
Scheduled Caste/Scheduled Tribe	0.341	0.335
	[0.475]	[0.473]
Other backward castes	0.501	0.502
	[0.501]	[0.501]
Pukka house	0.386	0.373
	[0.487]	[0.485]

*Notes*: Table reports variable means and standard deviations.

## 4 Measuring agency with three types of data

### 4.1 Quantitative surveys

We administered a 45-min survey that asked close-ended questions to the full sample of 443 study participants. It was conducted by female enumerators.

After asking a few questions on demographic characteristics such as age and religion, the questionnaire focused on measures of women’s agency within her household. We asked a long list of such questions, aiming to be exhaustive. We drew on existing questions to measure instrumental and intrinsic agency from other surveys. These included questions from the Demographic and Health Surveys, Relative Autonomy Index (Ryan and Deci, 2000, Vaz et al., 2016), a J-PAL toolkit on measuring women’s agency that aggregated survey questions that were used in several research studies (Glennerster, Walsh, & and Diaz-Martin, 2018), and the Sexual Relationship Power Scale (Pulerwitz, Gortmaker, & DeJong, 2000). We also included a handful of questions that we developed ourselves.

Concatenating all of the existing modules would introduce a lot of redundancy, resulting in a long and repetitive survey from the respondent’s point of view, so we made judgment calls in removing questions that overlapped. In total, we asked 63 questions measuring agency. The question order was not randomized. (The list of questions is provided in Appendix B.).

Some of the agency questions were about the woman’s say in specific decisions, such as, “If money is available, who in your household decides whether to pay school fees for a relative from your side of the family?” and “Can you go unescorted to the next village?” Other questions were more general, asking the woman about her overall impression of her agency. An example is, “This is a ten step ladder, where on the bottom, the first step, people who are completely coerced or powerless stand, and on the highest step, the tenth step, stand those with the most ability to advance goals that they value in their own homes and in the world. On which step are you today?”.

We convert each of the survey responses to a single numerical variable. Some of the responses have a natural numerical unit (e.g., days) or are binary. For questions asked on a Likert scale, we treat the categorical response as a cardinal variable. In a handful of cases where the numerical mapping is less clear, we make judgment calls. For example, in questions asked about whether women make decisions alone, jointly with their husband, or not at all, we code those responses as 2, 1, and 0. Note that we code all of the variables so that a higher value corresponds to more agency.^10^

It is also possible to include multiple variables, or recodings, per survey question; the important constraint is the number of survey questions at the data collection stage, not the number of variables. For the ladder question mentioned above, we could construct variables for the response being ⩾2, being ⩾3, and so forth up to the response equaling 10, or we could be agnostic about whether a woman having sole decision-making power represents strictly greater agency than joint decision-making with her husband. This approach would use more information and allow the data to determine the best recodings. We use one variable per survey question in our main index for simplicity but note that one of the statistical algorithms we use (random forest) considers all possible recodings.

### 4.2 Semi-structured interviews

The semi-structured interviews were on average 45 min long. They were conducted primarily by two female interviewers who had prior experience with in-depth interviewing. A third interviewer conducted a few of the interviews. As part of their training, one of the authors (MB) observed each interviewer conducting pilot interviews and provided feedback to improve their interview skills. The interviewers and MB met weekly to discuss substantive and methodological issues that arose, with learnings fed back into subsequent interviews.

The interviews, which were recorded, followed an interview guide (see Appendix C) that was refined through piloting. The initial guide covered five domains of agency within the household: the respondent’s decision-making around her children’s education and health, household expenditures, and her own fertility and mobility. In pilot interviews, employment emerged as another theme and was added as a sixth domain to probe in the interview.

The interviewers were trained to follow the interview guide and cover all six domains but to use their judgment to phrase questions differently, ask follow-up questions, or otherwise diverge from the guide if they felt that doing so would elicit better information from the respondent. The open-endedness of the interviews and the multiple domains allowed women to discuss direct and hidden strategies and the meanings behind their actions, including “bargaining and negotiation, deception and manipulation, subversion and resistance, and more intangible, cognitive processes of reflection and analysis” (Kabeer, 1999, p. 438).

To ensure privacy during the interviews, we paired each interviewer with someone initially recruited for our quantitative surveyor team who acted as a “distractor.” The distractor would have a discussion with other family members in a separate room so that the qualitative interviewer and study participant could have an uninterrupted private conversation.

The interviews were transcribed, and two people, the same two who conducted the interviews, coded them using Dedoose software. We randomly assigned which interviews each person coded, so in about half the cases, it was an interview she had conducted.

We used a two-step approach to coding, following Deterding and Waters, 2018. The first step in their “flexible coding” process is the development of “index codes” to represent the broad topics pursued during the research. In this study, the index codes were the six domains of agency that the interview focused on. The second step is the application of “analytic codes,” which emerge in the second reading of the transcripts. We paid attention to “speech practices” in our transcripts following Madhok, 2014, since agency is often more than observable action, and women’s own words open up the range of possibilities of what they consider agentic in their particular context.

The analytic codes were used to arrive at ranks (i.e., scores) and ranking definitions for each index code. The use of qualitative data to arrive at numeric scores has been widely used in participatory rural appraisal (PRA) methods (Chambers, 1994, Shaffer, 2013). MB and the coders triply coded and then discussed ten transcripts to harmonize how the coders interpreted and applied the codes. It was important that the scoring was done by the same people who had conducted the interviews because they were closest to the data, were from the region and were qualitatively trained.

The ranks ranged from 1 for a woman with the lowest level of agency to a 4 for a woman with the highest level of agency.^11^ As an example of the ranking definition and how the analytic codes map to the definitions, in the mobility domain, a woman coded as a 1 needs explicit permission to leave the house and always goes accompanied by her husband or someone else to locations either inside or outside the village, which includes the neighborhood store, her children’s school, the hospital, the market, the bank and her natal village. If a woman has those restrictions but objects to them or sometimes tries to resist them, she is coded as a 2. That is, if the analytic codes “never goes alone” but also “resistance” were coded in the transcript under the index code “mobility,” the woman’s rank moved from 1 to 2. A woman who has some but not all of the restrictions was coded as a 3; for example, she might be allowed to go to locations inside the village by foot, but is unable to go unaccompanied to locations that require transportation. Women with the most agency over their mobility were coded as a 4. They are able to go unaccompanied to all locations.

**Fig. 2:**
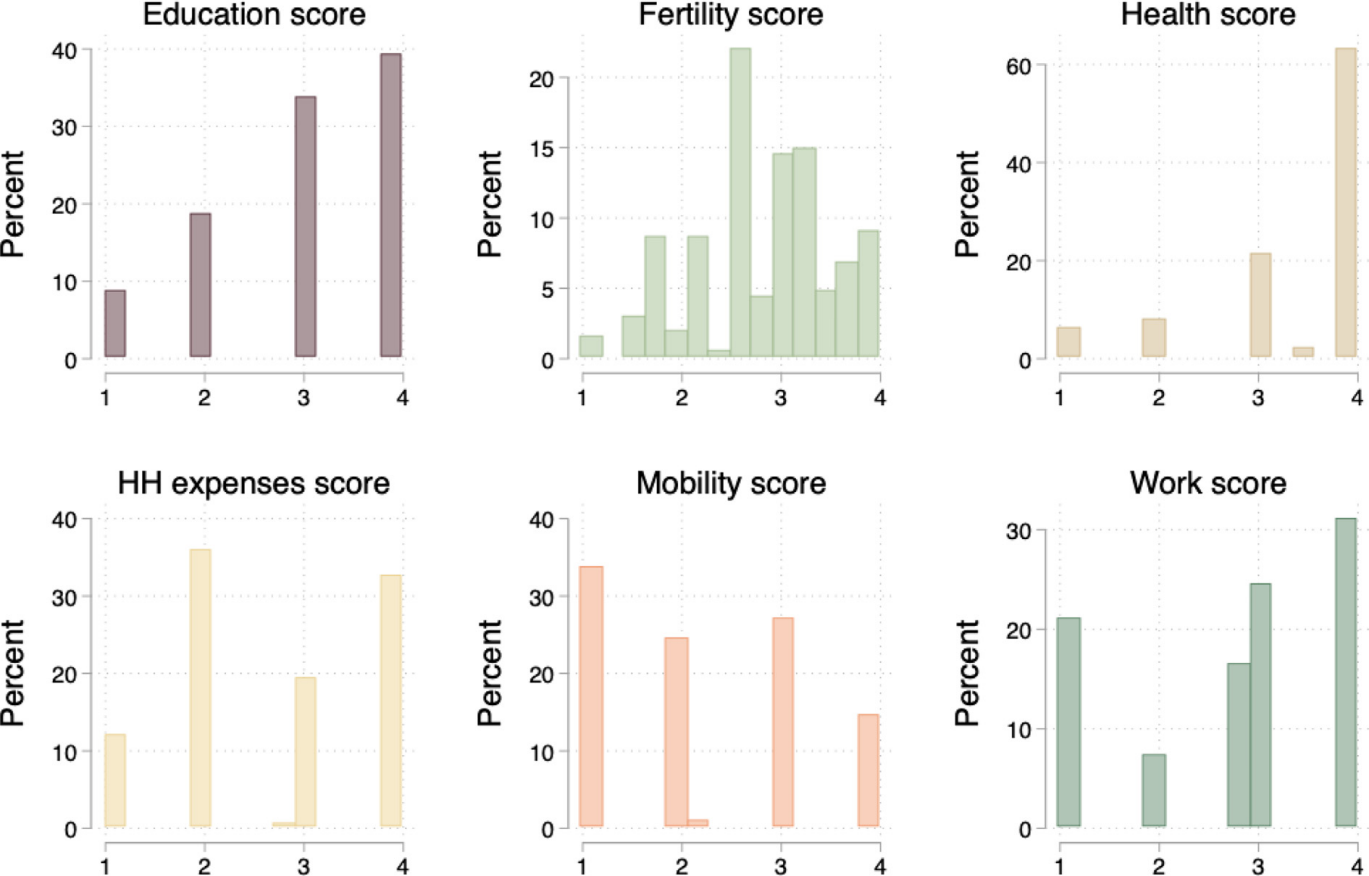
Distribution of scores from semi-structured interviews, by domain. *Notes*: The histograms show the scores for the six domains covered in the qualitative interviews. Fertility scores include non-integer values because the score averages across sub-domains. In other domains, non-integer values correspond to the 10 triple-coded surveys, for which we use the average scores across coders.

The one domain not initially coded on a 1 to 4 scale is fertility. Many women had discordant levels of agency across the four sub-domains of number of children, birth spacing, reversible birth control, and sterilization, so we coded a woman separately in each of the sub-domains and then averaged these scores. This fertility score was then re-scaled to also range from 1 to 4. Every domain had multiple questions, but when defining ranks, only the questions in the fertility domain required that sub-questions be mapped individually. In the example above on mobility, there were multiple questions on specific locations, but it was not necessary to map responses to locations separately because what mattered is whether women needed permission and needed accompaniment in general. Fig. 2 shows histograms of the domain-specific scores.

**Fig. 3:**
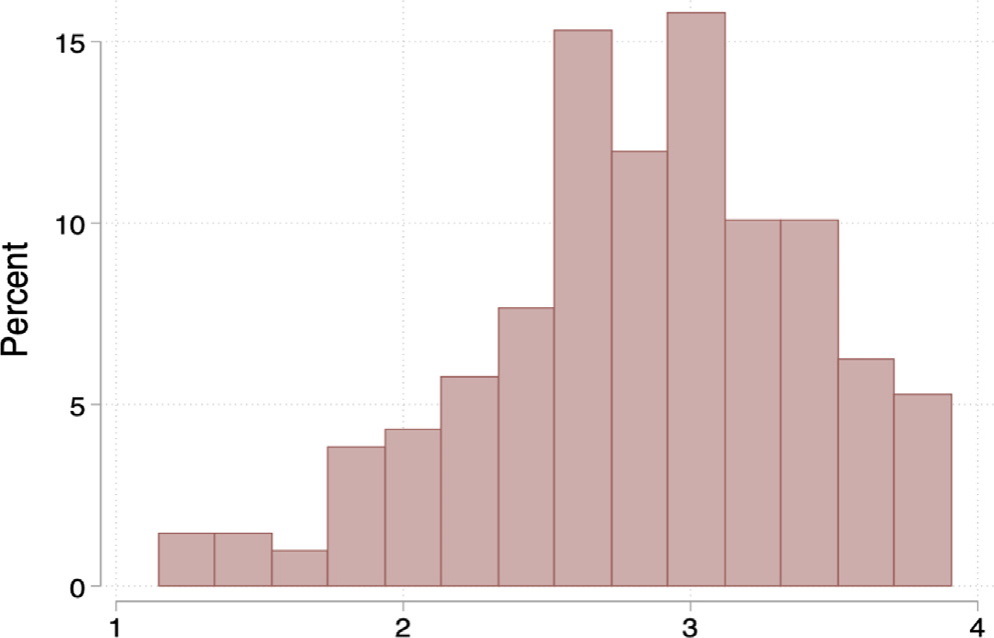
Distribution of overall scores from semi-structured interviews. *Notes*: The histogram shows the overall qualitative agency score for women in the sample, which is the simple average of her scores in the six domains. *Notes*: The figure is a histogram of women’s crossover point in the lab game, or the maximum amount they would forgo for their household to be the recipient of the money. A woman whose WTP is ₹ 400 prefers ₹ 300 for herself to ₹ 700 for her husband. A negative WTP means the woman prefers money to go to her husband, all else equal, e.g., -₹ 200 means that a woman prefers ₹ 100 for her husband over ₹ 300 for herself.

We then calculate an overall agency score for the woman as the average across the six domains.^12^ Fig. 3 shows the distribution of the overall agency score, as coded from the semi-structured interviews. Hereafter, we refer to the overall score as the qualitative score.

### 4.3 Lab game

We also used a lab-in-the-field game to measure women’s agency over household income. The game was conducted during the same visit and by the same surveyor as the quantitative survey. It took place in private at the end of the survey and took on average 15 min.

The measure uses real-stakes choices the woman makes, specifically her willingness to pay (WTP) to be the recipient of money given to the household. This measure was developed by Almås et al., 2018 in a study in urban Macedonia and has since been used in other settings, including Zambia and Tanzania (Barr et al., 2020, Almås et al., 2020). A potential advantage of a real-stakes choice is that it provides an objective, quantitative measure of the woman’s behavior. Because money is at stake, a respondent might be less subject to experimenter demand effects through which she gives insincere answers.

In the game, the woman is offered choices between ₹ 300 (4 USD) for herself and different amounts of money to be given to her husband.^13^ We inform her that one of her choices will be chosen at random and actually implemented, which gives her an incentive to report her true preferences (Becker, DeGroot, & Marschak, 1964).

As Almås et al., 2018 explain, in a unitary household, that is, if the husband and wife have identical preferences or are perfectly altruistic toward each other, women should try to maximize the transfer amount. But, the authors write, “in a non-unitary model, the weaker the position of the woman in the household (the lower her control of resources), the more she should be willing to pay to obtain control of that transfer.” Thus, some women might prefer ₹ 300 for themselves over ₹ 700 for their husband because they would have so little say in how their husband’s money is spent. A woman’s WTP to be the recipient of the money is the maximum amount she would forgo in total household income to be the recipient. The premise of the game is that the higher her WTP, the lower is her agency.

**Fig. 4:**
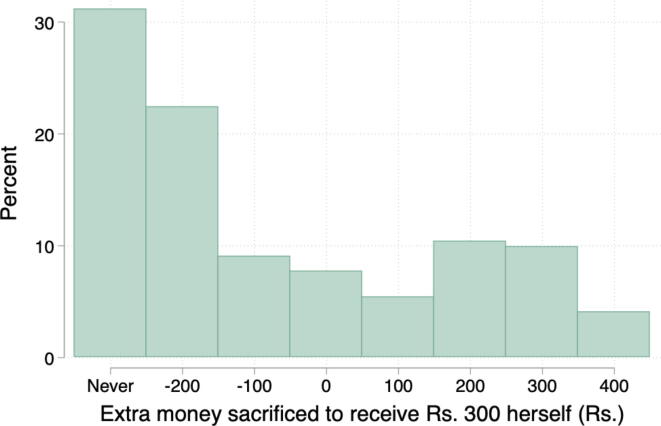
Distribution of women’s WTP to be recipient of money in lab game. *Notes*: The figure is a histogram of women’s crossover point in the lab game, or the maximum amount they would forgo for their household to be the recipient of the money. A woman whose WTP is ₹ 400 prefers ₹ 300 for herself to ₹ 700 for her husband. A negative WTP means the woman prefers money to go to her husband, all else equal, e.g., -₹ 200 means that a woman prefers ₹ 100 for her husband over ₹ 300 for herself.

This reasoning implies women should have a positive WTP to control the money, with perhaps some highly empowered women have a WTP of zero. However, in our context, many women always preferred that their husband get the money even when it was less than ₹ 300 and thus they had a negative WTP. Fig. 4 shows the distribution of WTP in our sample.

We debriefed with women who had a negative WTP to understand their behavior in the game (Jackson, 2011). This revealed that their choice was linked to having low agency; they believed that women should not get involved with household finances, or they feared that their husband would find out they received money. The theoretical premise of the measure is that low-agency women will have a higher demand for agency, but many women with low agency in fact did not want more agency. After noticing this pattern in the field and then seeing the distribution of WTP, we became pessimistic that using WTP as the benchmark measure would yield a reliable survey measure of agency.

## 5 Statistical algorithms to select survey questions

The goal of our data analysis is to choose the best five survey questions to measure women’s agency. We do so by selecting those that are the best predictors of a benchmark measure of agency.

An intuitive approach to finding the best subset of survey questions would be to try every possible combination of five questions and use the set that yields the highest $R^{2}$ in a linear regression in which the benchmark measure is the outcome and the survey variables are the regressors. A pitfall of such an approach is that it is subject to over-fitting. Machine learning algorithms typically leave out a portion of the data during estimation, and then adjust the algorithm parameters or estimates based on how accurate the predictions are in the left-out sample (e.g., cross-validation). In addition, an exhaustive search can be computationally infeasible (there are over 7 million ways to choose five variables from among 63). We thus apply two statistical algorithms (LASSO stability and random forest selection) that address over-fitting and are computationally feasible. We also use a third technique (backward sequential selection) that addresses computational feasibility and adds robustness through an iterative process, but does not cross-validate the prediction.

Standard supervised machine learning techniques like LASSO and random forest share our goal of out-of-sample prediction.^14^ The distinction here is we want to put a rigid constraint on the number of predictors to select. If standard LASSO chooses 15 variables, that would yield a survey module that is impractical for many purposes. The three statistical algorithms we implement, described below, aim to identify the five most valuable questions. This type of analysis is referred to as feature selection in the machine learning literature.

Below we first describe LASSO stability selection, which is our preferred approach; it strikes a balance between simplicity and robustness. The second algorithm builds on random forest and is more complex, while the third algorithm, backward sequential selection, is the simplest one. At the end of this section, we compare the algorithms in more detail.

### 5.1 LASSO stability selection

In the LASSO stability selection algorithm, the best questions are those most commonly selected when LASSO is repeatedly run on subsamples of the data. Meinshausen and Bühlmann (2010) show that variable selection through this combination of regularized regressions (e.g., LASSO) and resampling (e.g., drawing subsamples) is quite robust to the choice of the tuning or regularization parameter.^15^

We use 50% subsamples and run LASSO 1000 times:^16^

1. Draw a 50% subsample of observations without replacement.
2. Run a LASSO regression of the benchmark measure of the outcome on all of the survey variables, keeping track of which predictors are selected, i.e., have coefficients not shrunk to 0.^17^
3. Complete 1000 iterations of steps 1 and 2.

The proposed survey module consists of the five survey questions chosen most frequently by LASSO across the iterations. We then combine them into an index by normalizing each of the variables to have a standard deviation of 1 and mean of 0 and averaging the standardized variables. We refer to this type of aggregation as a standardized index. Using (regular or LASSO) regression coefficients as weights to create a weighted index is another natural way to combine the variables. We opt for just an average of the standardized variables for simplicity and to make the aggregation less dependent on the estimates.

Unlike in some prediction exercises, there is a “correct” sign of each regression coefficient in our case. The premise of our criterion validation exercise is that we are regressing one measure of agency on another, so the sign of the coefficients should be positive. Nothing in the statistical procedure constrains the coefficients to be positive. Thus, one diagnostic for how well the procedure works is whether any of the coefficients are wrong-signed.

### 5.2 Random forest selection

The second algorithm we use is Genuer et al.'s (2010) variable selection using random forest, or VSURF, algorithm. The basis of this algorithm is random forest, which classifies data using decision trees.^18^ VSURF entails building a series of random forests, first to narrow the variable set based on a variable importance metric and then to compare random forests that use different variable subsets to identify the variables with the most predictive power.^19^

This algorithm is considerably more complicated than the other two we implement. A reader who is not interested in the technical details can skip the rest of this subsection.

The algorithm proceeds as follows:

1. Build 100 random forests using all of the available predictors. Calculate the average across the forests of each variable’s variable importance (VI), which is a measure of the improvement in model prediction when one includes the variable.^20^ Retain a variable if the standard deviation of its VI across the 100 forests exceeds a threshold.^21^
2. Build 100 random forests using the most important variable from step 1, then 100 random forests using the two most important variables, and continue up to 100 random forests using all variables retained in step 1. From among these models (where each model is an average of 100 forests), retain the smallest one (i.e., fewest variables) among those with an out-of-bag (OOB) error less than a threshold.^22^
3. Build another set of random forest models, sequentially introducing the variables retained after step 2, in the order of VI from step 1. Build and average 100 random forests that include the introduced variable. Keep the variable in the model if it decreases OOB error, relative to the model thus far, by more than a threshold amount.^23^

We tune the threshold in the final step of the algorithm so that the desired number of variables (five) are selected.^24^

### 5.3 Backward sequential selection

The third algorithm we use is a simplified version of a backward sequential selection technique using linear regression (Liu & Motoda, 1998). The general algorithm — iteratively removing the least important variable — is often referred to as recursive feature elimination (Guyon, Weston, Barnhill, & Vapnik, 2002).

We start with the full set of survey questions and iteratively remove the one that adds the least predictive power (for predicting the benchmark measure), stopping when the target number of questions (in our case, five) are left.^25^ At each step, we could assess the $R^{2}$ of multivariate regressions of the qualitative score on the candidate variables. Because ultimately most researchers will want to use the selected variables to construct an index, we combine them into an index at the selection stage. At the iteration with *k* variables left, for all combinations of k-1 of them, we combine the variables into a standardized index and estimate a univariate regression of the benchmark measure of the outcome on the index; equivalently, the assessment is based on the correlation between the benchmark value and the index.

The first step is to combine all the candidate survey variables on agency into an index. Then we iteratively remove variables as follows:

1. Discard one of the available variables and combine the remaining *k* variables into an index (after normalizing them).
2. Calculate the correlation coefficient between the benchmark measure of agency and the index.
3. Repeat steps 1 and 2 for all remaining variables.
4. Drop from the set the variable that led to the smallest decrease (or largest increase) in the correlation coefficient, relative to including all *k* in the set.
5. Repeat steps 1 to 4 until the desired number of variables for the index is reached.

The last five questions that remain comprise the proposed survey module, and the standardized index based on them is the proposed measure of women’s agency.

Note that we do not include any cross-validation in the algorithm, although in principle one could.

### 5.4 Comparison of the three algorithms

Our rationale for using three different algorithms was to better understand how sensitive the general approach — combining machine learning and qualitative interviews for survey design — is to the specific statistical algorithm used.

LASSO stability selection and random forest selection both address over-fitting in each iteration or decision tree. An advantage of the LASSO approach is the final model’s transparency or interpretability. The model prediction is a parsimonious five-term linear equation. For random forest, the model prediction is an average across many trees of many interaction and non-linear terms. Moreover, the “wrapper algorithm” around LASSO used in LASSO stability selection is simple iteration, while the VSURF (random forest) wrapper algorithm is more complex. Thus, LASSO stability selection’s attractiveness relative to random forest selection is the transparency of the algorithm and the resulting model.

Backward sequential selection’s disadvantage is that, in our implementation of it without cross-validation, it does not address over-fitting. Its advantage is its simplicity: It uses a standard linear regression in each iteration.

For each of the algorithms, we propose to combine the five variables into a standardized index. The algorithms differ in how restrictive this method of aggregation is. Backward sequential selection optimizes the predictive power of the top five questions when they are combined in this way; there is no mismatch between the predictive model and how the selected questions are then aggregated. LASSO stability selection collapses each question to a linear variable, which matches how the questions are then aggregated. However, the top variables are chosen without their aggregate predictive power taken into consideration. Two highly ranked variables could be collinear and thus redundant, with each chosen in different LASSO iterations. (This does not occur in practice in our application). Aggregating via a standardized index is the least appropriate for random forest. The advantage of random forest is that it allows for non-linearities and interaction terms, but the aggregation then discards this information. Thus, when we present the results, we also consider the predicted value from the model as an alternative index. For random forest, this alternative index has a much stronger correlation with the benchmark measure.

Putting this all together, we favor LASSO stability selection among the algorithms because it addresses over-fitting yet is transparent and intuitive. Backward sequential selection is a potentially useful alternative because it involves nothing more than a loop over ordinary linear regressions. Random forest can extract more information from five variables, so it might be the first choice of researchers who are undeterred by a more complex algorithm and index.

## 6 Results: Validated survey module for women’s agency

### 6.1 Based on semi-structured interviews as benchmark

We report the best set of survey questions to measure agency, as determined by the MASI method, in Table 2. These are the questions chosen based on their correspondence with the qualitative score.

**Table 2:** Selected survey questions using semi-structured interviews.

Question	LASSO stability selection	Random forest selection (VSURF)	Backward sequential selection
	(1)	(2)	(3)
Opinion heard when expensive item like a bicycle or cow is purchased?	1	3	2
Need permission from other household members to buy clothing for self?	2		1
Allowed to buy things in the market without asking partner?	3	2	
Are you permitted to visit women in other neighborhoods to talk with them?	4	4	4
Who do you consult with for decisions regarding your children’s health care?	5		
Are you permitted to visit any place riding on public transport?		1	
Who in household decides to pay school fees for a relative from your side of family?		5	5
Allowed to go alone to meet your friends for any reason?			3

5-question standardized index corr. coeff.	0.537	0.501	0.535
5-question model prediction index corr. coeff.	0.538	0.784	0.538

*Notes*: The table lists the top 5 survey questions selected. (See Appendix B for the full question wording.) The numbers in the cells in columns (1) to (3) indicate the selection order, with 1 referring to the best, or most predictive question. The reported correlation coefficients are between the qualitative score and the index.

Table 2, column (1) reports the questions selected using LASSO stability selection. The numbers in the cells are the rank for the question, in terms of how often it was selected in LASSO iterations estimated on subsamples of the data.^26^ The top question is about decision-making regarding large household purchases like a cow or bicycle. The variable was selected in 85% of the LASSO iterations, as reported in Table 3. The fifth question was selected 58% of the time. Table 3 provides the frequency of selection for the top ten variables; if a researcher seeks a ten-question module, these are the best choices based on the algorithm. The fourth- to sixth-ranked questions perform fairly similarly to each other, and the biggest gains from the algorithmic approach seem to be from identifying the best three questions. The lowest-ranked of the 63 candidate questions was selected in 2% of the LASSO iterations.

**Table 3:** Frequency of variable selection using LASSO stability selection.

Question	Percent of times selected
Opinion heard when expensive item like a bicycle or cow is purchased?	84.9
Need permission from other household members to buy clothing for self?	76.4
Allowed to buy things in the market without asking partner?	73.8
Are you permitted to visit women in other neighborhoods to talk with them?	59.3
Who do you consult with for decisions regarding your children’s health care?	58.1
Are you permitted to visit any place riding on public transport?	57.6
Allowed to go alone to meet your friends for any reason?	54.8
Can decide by self to purchase emergency medicine for child	52.3
Are you allowed to go alone to a relative’s house inside the village?	47.4
When husband has different opinion, voice opinion and argue more often than voice opinion but do as he says*	47.3

*Notes*: The numbers reported are how often, out of 1000 iterations of LASSO on 50% subsamples, a variable was selected as a regressor in the LASSO stability selection procedure. The dependent variable is the semi-structured interview score. * This variable is constructed from a series of separate questions. See Appendix B for more details and for the full wording of the questions.

Interestingly, none of the general questions that ask a woman to assess her overall agency or perception of her power are among the top questions. The top three questions ask about her role in specific purchase decisions: large household purchases, clothing for herself, and items in the market. The other two questions pertain to her physical mobility (whether she can visit women in her neighborhood without permission) and to decisions about her children’s health care. The mobility question highlights that the best five-question module is likely to differ by context; restrictions on women’s travel within their village are more common in north India than many other places (Rahman and Rao, 2004, Jayachandran, 2015, Naybor et al., 2016).

All five of the selected variables are predictive in the correct direction; with the variables coded such that a higher value theoretically represents more agency, the raw correlation with the qualitative score is always positive. Appendix Table A.1 shows the correlation between the qualitative score and each of the selected variables.

The proposed way to combine the survey questions into one measure is to average the five variables: We code each survey question as a continuous variable, make them comparable by normalizing each to have a standard deviation of 1, and then average them. The correlation coefficient (*r*) between the qualitative score and the resulting index is shown at the bottom of Table 2. Using the LASSO-stability-selected questions (column 1), r=0.54. The next row shows the correlation coefficient if we instead use the model prediction as an index, specifically the predicted value of a LASSO regression of the qualitative score on the five variables; one does not lose much information by using the standardized average. This simple way of aggregating, therefore, seems suitable for many purposes.^27^

Appendix Table A.2 shows the correlation between the survey index and qualitative scores in each of the six domains. The index is most strongly correlated with the household expenditures and mobility domains, which is unsurprising as four of the five selected questions are within those two domains.

We now turn to the results using the two other statistical algorithms. Table 2, column (2) reports the top five questions selected using random forest selection. Three of the questions are in the set chosen by LASSO stability selection, though not in the same order.^28^ The new variables that are selected pertain to household spending and mobility. For the qualitative score and a standardized index of the top random forest variables, r=0.50. It is unsurprising that random forest performs worse than LASSO stability because, in averaging the five variables, we are ignoring the non-linearities and interactions that random forest selection allowed for when identifying the best variables.

It is also informative to assess random forest selection when using the model’s predicted value as the women’s agency index. We take the five selected variables, build a random forest using them, and extract the predicted value for each observation. Here, random forest performs much better than LASSO stability selection; its model prediction is more strongly correlated with the qualitative score than is LASSO stability selection’s. This is again unsurprising: Random forest allows for more degrees of freedom when using the five variables as predictors. A researcher could choose to use the random forest set of questions and then estimate a random forest model with her data to extract the predicted value as the women’s agency index or use the predicted value from the random forest trained on our data.^29^ The resulting index would be a richer but more black-box measure.

In Table 2, column (3), we report the top questions based on backward sequential selection. Three of them overlap with the set chosen by LASSO stability selection, and three overlap with the random forest set. The new variables are related to household spending and mobility. For the index based on the backward sequential selection questions and the qualitative score, r=0.54, almost identical to what was found for the LASSO stability selection index. It is somewhat surprising — and reassuring — that LASSO stability selection, which chooses variables taking into account out-of-sample fit, achieves as much within-sample predictive power as backward sequential selection.

### 6.2 Comparison to randomly choosing variables

**Fig. 5:**
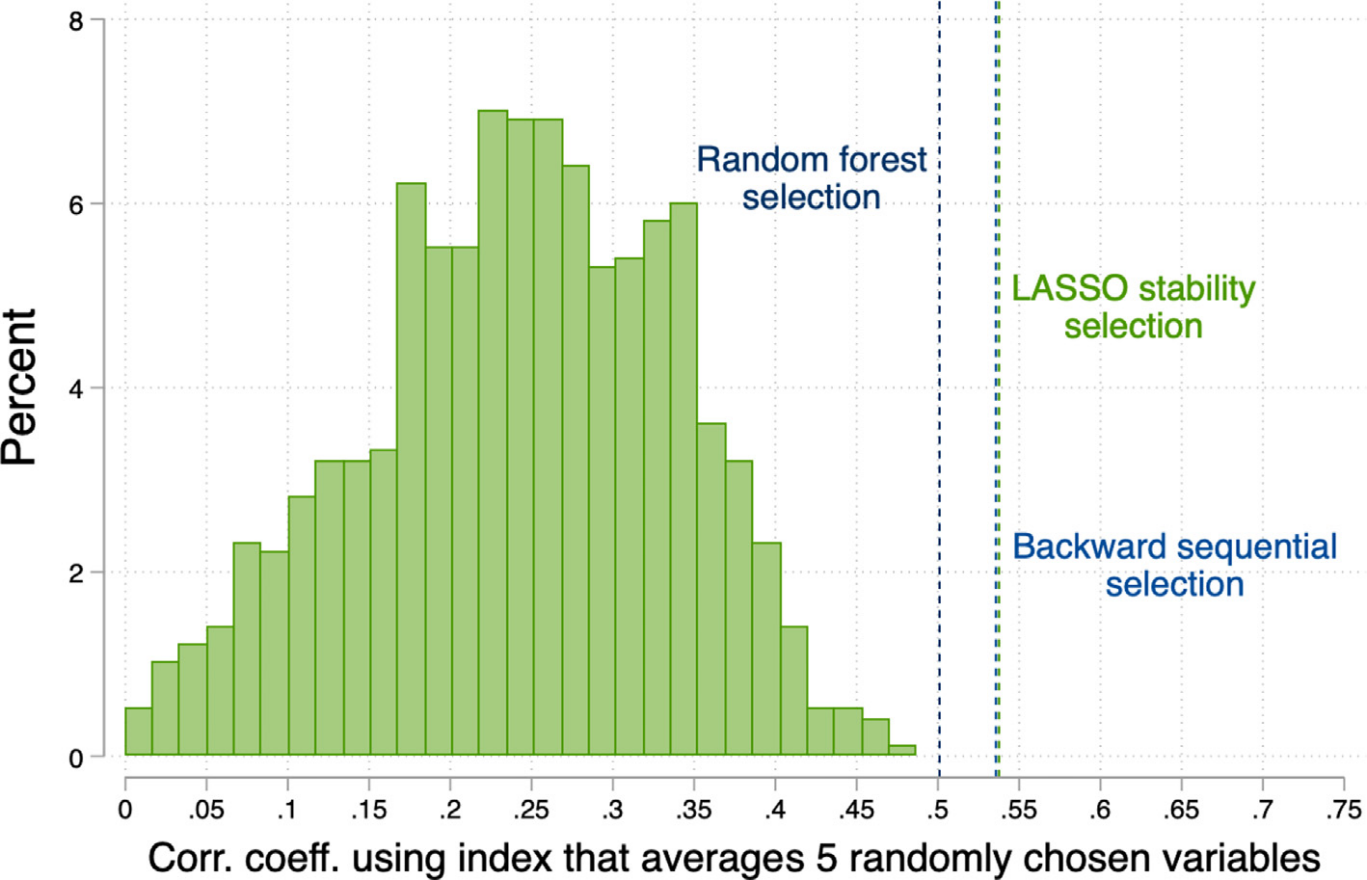
Selected indices compared to five randomly chosen variables. *Notes*: We take 1000 random draws of 5 out of 63 questions. The figure plots the distribution of the correlation coefficient between the qualitative score and a standardized index combining the 5 variables.

One way to gauge how valuable it is to use an algorithmic approach to survey question selection is to compare it to ad hoc selection. Fig. 5 plots a histogram of index performance, specifically the correlation coefficient between the qualitative score and the index, if we randomly select five questions from among the 63 candidates. The median *r* across 1000 randomly selected sets of variables is 0.25. The three algorithm-selected indices do considerably better than not just the median, but also the 99th percentile of the distribution using randomly selected variables.

### 6.3 Comparison to LASSO

When we estimate standard LASSO using the qualitative score as the dependent variable and the 63 candidate survey variables as potential regressors, LASSO selects 15 regressors (which are listed in Appendix Table A.3). Reassuringly, among them are all 8 survey questions that are in the top 5 set for one or more of the statistical algorithms, which need not have been the case.

If all of the LASSO-selected variables are combined into a standardized index, r=0.60. Using the predicted value of the LASSO regression as the agency index, r=0.61. These correlations are higher than one obtains with the five-question indices, but come at the cost of a longer (fifteen-question) survey module. We return to this trade-off between performance of the index and brevity later in this section.

### 6.4 Comparison to using all 63 close-ended survey questions

Another benchmark is if we constructed an index using information from all 63 variables. The $R^{2}$ of a multivariate regression of the qualitative score on all of the variables is 0.51. In the counterpart regression of the qualitative score on the five-question LASSO stability selection index, $R^{2}=0.29$. One sacrifices less than half of the explanatory power when using only 5 out of 63, or 8%, of the potential survey questions, and combining them into one measure.

Averaging all 63 variables in a standardized index actually leads to a lower correlation with the qualitative score (r=0.46) than one achieves using the five-question indices. The cost of using more variables is not just that it requires a longer survey, but also that some variables are weak (or wrong-signed) predictors of agency as measured by the qualitative interview, so including them lowers the predictive power of the index.

Another common way to create an index based on multiple variables is through principal component analysis. If one uses the first principal component of the 63 variables as the measure of agency, r=0.48, which is again lower than what the algorithms achieve.

### 6.5 Trade-off between the length and performance of the survey module

The fact that an index using all 63 survey variables performs worse than using the five selected variables raises the question of how index performance is related to the number of variables selected. We repeated the three algorithms incrementing the number of selected variables from 1 to 63. Appendix Fig. 6 plots the predictive power of the selected indices. For LASSO stability selection, the *r* peaks at 0.59, with the best 19 questions included. Recall that using the best 5 questions, r=0.54. The maximum *r* is achieved with 13 questions and 16 questions using random forest selection and backward sequential selection, respectively.

**Fig. 6:**
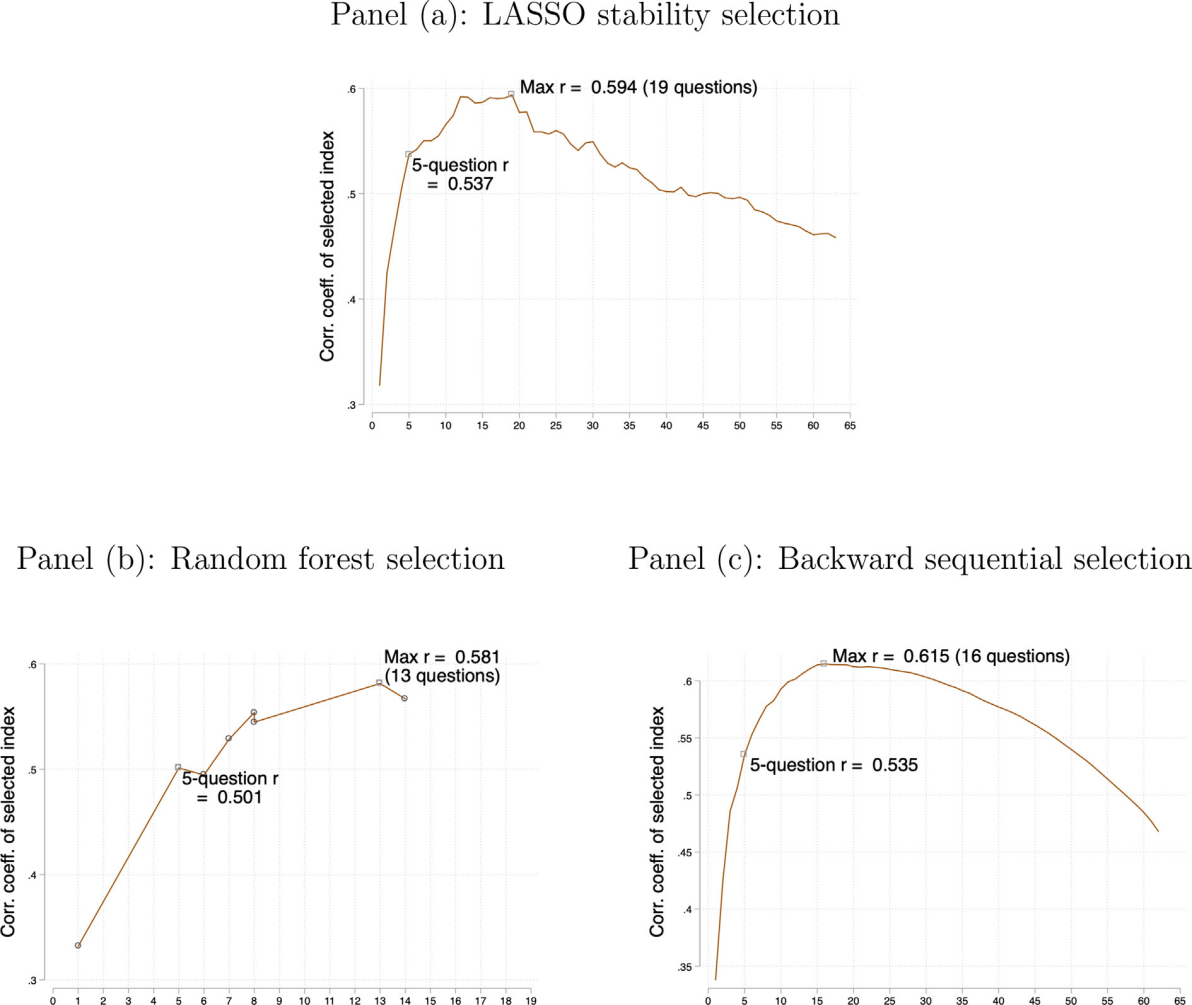
Performance of the indices when the number of questions is varied. *Notes*: The figures plot correlation coefficient (*r*) between the qualitative score and a standardized index constructed from the best *k* variables selected by the algorithm; the value *k* is plotted on the horizontal axis. LASSO stability selection produces a ranked list of all variables (as all variables are selected in some LASSO iterations in our application); thus an index is produced for each value of *k* from 1 to 63. Backward sequential selection also ranks all variables. For random forest, we vary the tuning parameter in the last step of the algorithm, which produces models with different values of *k* but not for all *k*. The maximum *k* shown in panel (b) is 14 because that is the maximum number of variables retained before the last step of the random forest algorithm across all possible values of the tuning parameters that influence earlier steps of the algorithm.

Thus, there is a trade-off between a shorter survey module and an agency index that captures more information, up to a point. A researcher willing to use a longer module could take the best 10 or 15 questions instead of the best 5 that we have focused on. But what is also apparent is that after a point, even if fielding a longer survey were not costly, using a larger number of agency variables in the index seems to hurt performance.

### 6.6 Correlation with characteristics often associated with women’s agency

As another assessment of the indices, we report their correlation with factors often associated with agency. For example, one might expect younger women to have less agency. Also, agency is often believed to be negatively correlated with the age gap between the husband and wife (that is, women who are considerably younger than their husbands have less agency), and likewise with the husband-wife education gap. A first step is to check the correlation between these factors and the qualitative score itself. As reported in Appendix Table A.2, the qualitative agency score is indeed positively correlated with the woman’s age and negatively correlated with the husband-wife education gap. In turn, the indices chosen by the three algorithms have the same-signed correlations with age and the education gap. Surprisingly, both the qualitative score and the three indices have a small positive correlation with the husband-wife age gap.

### 6.7 Sensitivity of the selected questions to interviewer and coder identity

The fact that the interview is conducted by a specific individual and one person coded the interview adds subjectivity to the qualitative agency score. To assess how much the algorithm-selected survey questions depend on the identity of the interviewer and coder, we repeated the analyses using only the data from one qualitative interviewer or one coder.

Appendix Table A.4, Table A.5, Table A.6 show the overlap in questions selected when we use the full sample or a specific interviewer or coder. As summary measures of robustness, Appendix Table A.7, Table A.8 report the correlations across the resulting indices. For example, for LASSO stability selection, the correlation between the main index and the indices based on a single interviewer are 0.85 and 0.77, and the correlation between the two interviewer-specific indices is 0.52. For random forest selection, the two interviewer-specific indices have correlations of 0.89 and 0.85 with the main index and a correlation of 0.88 with each other. When we compare the coders, the correlations between the coder-specific indices and the main index are 0.95 and 0.74 for LASSO stability and 0.83 and 0.84 for random forest. The correlation between the two coder-specific indices is 0.63 for LASSO stability and 0.61 for random forest.^30^

We view this degree of correspondence across interviewers and coders to be high. Because we are analyzing subsamples, there is more sampling error than when using the full sample (as discussed below). Thus, even if there were no true interviewer or coder effects on the qualitative score, the overlap in selected questions in this analysis would be imperfect.

### 6.8 How well would MASI have performed with a smaller sample size?

A sample size of 209 qualitative interviews might be impractically large in some applications, due to time or budget constraints. To understand how well MASI would work with a smaller sample size, we drew random subsamples of 100 observations (48% subsamples) and repeated the variable selection process, focusing on the LASSO stability selection algorithm. We repeated this 100 times and assessed how well the 100 resulting indices performed and the degree to which the selected questions overlapped with those chosen with the full sample.

The top full-sample question, about the woman’s say in large household purchases, is among the top 5 selected questions 73% of the time when we use 100-observation subsets of the data. On average, 2.4 questions from the full-sample set of five questions were selected using the smaller samples. Another metric for assessing performance is the correlation between the resulting indices and the qualitative score. The average correlation using the smaller subsamples is 0.48; the correlation is 0.54 for the index created using the full sample.

To summarize, there is some instability in the specific questions chosen if one uses a smaller sample size. However, much of the value of MASI seems to derive from identifying the best one or two questions plus the next six to ten very good questions. A smaller sample size seems to suffice for these purposes.

### 6.9 Based on lab game as benchmark

Given the problems with the lab game discussed in Section 4.3, it is unsurprising that survey indices created by using the lab game as a “true” measure of agency do not perform well. For completeness, we report the selected questions in Appendix Table A.12.^31^ One indication that the questions validated against the lab game are less reliable is that the index combining them is not strongly correlated with the lab game measure of agency (r=0.21 using LASSO stability selection, for example). Moreover, the top question from LASSO stability selection is selected in only 18% of the LASSO runs. Also, two of the top questions based on random forest selection have a negative (i.e., wrong-signed) correlation with the lab game measure of agency. These results reinforce our conclusion that the lab game was an inadequate tool for measuring women’s agency — and thus for applying MASI — in our study.

## 7 Conclusion

In this study, we developed a new five-question survey module for women’s agency from a starting set of 63 questions, using a data-driven approach. This short module could be useful for those seeking an off-the-shelf way to measure agency in north India and perhaps elsewhere. The module was created using data from married women with children in one part of India, so a valuable direction for future research is to replicate the study in other populations. Indeed, the fact that some of the selected questions pertain to women’s physical mobility, a dimension of agency particularly salient in India, highlights the context-specificity of women’s agency and its measurement (which is likely also true of other constructs studied in economics and other social sciences).

Another finding that highlights the importance of context is that behavior in a lab game that has been used in Macedonia, Zambia, and Tanzania mapped to agency in too messy of a way in our study to serve as a good benchmark measure. Specifically, the game uses high demand for agency as a proxy for having low agency, but many women with low agency did not want more agency. We conclude that using semi-structured interviews to obtain a benchmark measure of agency is advantageous in large part because such interviews are intrinsically context-specific, with the flow of the conversation adapting to the woman’s responses.

The primary contribution of the study is to introduce a new method for developing validated measures of constructs by combining machine learning and semi-structured interviews (MASI). Based on the principle of criterion validation, the method vets quantitative measures of a construct by benchmarking them against semi-structured interviews. Specifically, we use supervised machine learning techniques to select the best survey questions based on how well they predict the measure of agency obtained through in-depth but time- and skill-intensive qualitative interviews.

MASI has many other potential applications. For example, the best questions to measure changes in a woman’s agency, such as those caused by policy interventions, might differ from the best ones to measure a woman’s current agency (our focus). One could carry out a similar study to create a survey module optimized for measuring changes, with the data collection carried out at two points in time, and the statistical analysis centered around changes in responses. More broadly, combining machine learning and semi-structured interviews to develop short survey measures of complex constructs has many promising applications beyond women’s agency.

## CRediT authorship contribution statement

**Seema Jayachandran:** Conceptualization, Methodology, Formal analysis, Writing - original draft, Funding acquisition. **Monica Biradavolu:** Methodology, Investigation, Formal analysis, Writing - review & editing. **Jan Cooper:** Methodology, Investigation, Writing - review & editing.

## Declaration of Competing Interest

The authors declare that they have no known competing financial interests or personal relationships that could have appeared to influence the work reported in this paper.

## Figures and Tables

**Table A.1:** Correlation of qualitative score and selected survey variables.

	Qualitative agency score	Opinion heard when expensive item like a bicycle or cow is purchased?	Need permission from other household members to buy clothing for self?	Allowed to buy things in the market without asking partner?	Are you permitted to visit women in other neighborhoods to talk with them?	Who do you consult with for decisions regarding your children?s health care?	Are you permitted to visit any place riding on public transport?	Who in household decides to pay school fees for a relative from your side of family?	Allowed to go alone to meet your friends for any reason?
Qualitative agency score	1.000								
Opinion heard when expensive item like a bicycle or cow is purchased?	0.318	1.000							
Need permission from other household members to buy clothing for self?	0.338	0.192	1.000						
Allowed to buy things in the market without asking partner?	0.346	0.287	0.324	1.000					
Are you permitted to visit women in other neighborhoods to talk with them?	0.295	0.120	0.155	0.211	1.000				
Who do you consult with for decisions regarding your children’s health care?	0.218	−0.054	0.123	−0.006	0.119	1.000			
Are you permitted to visit any place riding on public transport?	0.332	0.194	0.278	0.369	0.369	0.124	1.000		
Who in household decides to pay school fees for a relative from your side of family?	0.176	0.019	0.143	0.206	−0.139	−0.013	0.145	1.000	
Allowed to go alone to meet your friends for any reason?	0.280	0.091	0.071	0.218	0.319	0.190	0.268	−0.018	1.000

**Table A.2:** Correlation of qualitative score, 5-question indices, and factors often associated with agency.

	Qualitative agency score	LASSO stability selection 5-Q index	Random forest selection 5-Q index	Backward seq. selection 5-Q index	Fertility score	Education score	Health score	HH expenses score	Mobility score	Work score	Age	Husband-wife age gap	Husband-wife education gap
Qualitative agency score	1.000												
LASSO stability selection 5-Q index	0.532	1.000											
Random forest selection 5-Q index	0.503	0.830	1.000										
Backward seq. selection 5-Q index	0.539	0.820	0.791	1.000									
Fertility score	0.346	0.197	0.143	0.233	1.000								
Education score	0.658	0.284	0.302	0.262	0.093	1.000							
Health score	0.634	0.281	0.221	0.321	0.153	0.409	1.000						
HH expenses score	0.707	0.436	0.429	0.438	0.093	0.355	0.367	1.000					
Mobility score	0.697	0.339	0.415	0.364	0.017	0.299	0.345	0.533	1.000				
Work score	0.479	0.322	0.232	0.283	0.131	0.170	0.046	0.083	0.157	1.000			
Age	0.218	0.187	0.187	0.177	−0.054	0.121	0.096	0.227	0.293	0.036	1.000		
Husband-wife age gap	0.071	0.032	0.016	0.034	−0.009	0.026	0.088	0.180	0.091	−0.122	−0.162	1.000	
Husband-wife education gap	−0.245	−0.075	−0.059	−0.141	−0.010	−0.170	−0.085	−0.235	−0.191	−0.137	0.055	0.054	1.000

**Table A.3:** All variables selected by regular LASSO (using semi-structured interview)

Question
Can decide by self to purchase emergency medicine for child
Who accompanied you to healthcare provider?
Who do you consult with for decisions regarding your children’s health care?
Opinion heard when expensive item like a bicycle or cow is purchased?
Allowed to buy things in the market without asking partner?
Who in household decides to pay school fees for a relative from your side of family?
Who in household decides purchasing item like radio or paraffin lamp?
Need permission from other household members to buy clothing for self?
Do you have a bank or savings account that you yourself use?
Are you permitted to visit any place riding on public transport?
Are you permitted to visit women in other neighborhoods to talk with them?
Are you allowed to go alone to a relative’s house inside the village?
Allowed to go alone to meet your friends for any reason?
When husband has different opinion, voice opinion and argue more often than voice opinion but do as he says
In last 12 months, how often you and husband discussed children’s expenses

*Notes*: The variables listed are the 15 ones chosen by standard LASSO when the dependent variable is the semi-structured interview score and the possible regressors are the 63 close-ended survey questions.

**Table A.4:** Selected survey questions using data from one interviewer or coder: LASSO stability selection.

Question	Main results	Interviewer 1 only	Interviewer 2 only	Coder 1 only	Coder 2 only
Opinion heard when expensive item like a bicycle or cow is purchased?	1	8	3	3	2
Need permission from other household members to buy clothing for self?	2	1		4	1
Allowed to buy things in the market without asking partner?	3	2	8	1	
Are you permitted to visit women in other neighborhoods to talk with them?	4		9	8	5
Who do you consult with for decisions regarding your children’s health care?	5	6			3
Are you permitted to visit any place riding on public transport?	6		1	5	
Allowed to go alone to meet your friends for any reason?	7		4	2	
Can decide by self to purchase emergency medicine for child	8	3			
Are you allowed to go alone to a relative’s house inside the village?	9	5		6	7
When husband has different opinion, voice opinion and argue more often than voice opinion but do as he says	10				

5-question standardized index corr. coeff.	.537	.467	.485	.521	.508
5-question model prediction index corr. coeff.	.538	.486	.493	.524	.52

**Table A.5:** Selected survey questions using data from one interviewer or coder: Random forest selection (VSURF).

Question	Main results	Interviewer 1 only	Interviewer 2 only	Coder 1 only	Coder 2 only
Are you permitted to visit any place riding on public transport?	1	1	10	2	3
Allowed to buy things in the market without asking partner?	2	5	1	1	
Opinion heard when expensive item like a bicycle or cow is purchased?	3	9			5
Are you permitted to visit women in other neighborhoods to talk with them?	4	3		7	4
Need permission from other household members to buy clothing for self?	5		5	4	
When was the last time you were unwell and visited a healthcare provider?	6				
In last 12 months, how often you and husband discussed children’s expenses	7			8	10
Are you allowed to go alone to a relative’s house inside the village?	8		3		
Allowed to go alone to meet your friends for any reason?	9	4		3	
Can decide by self to purchase emergency medicine for child	10		4	10	

5-question standardized index corr. coeff.	.501	.461	.467	.49	.444

*Note*: This analysis uses only step 1 of the VSURF procedure, i.e., the ranking based on the variable importance metric.

**Table A.6:** Selected survey questions using data from one interviewer or coder: Backward sequential selection.

Question	Main results	Interviewer 1 only	Interviewer 2 only	Coder 1 only	Coder 2 only
Need permission from other household members to buy clothing for self?	1	1			4
Opinion heard when expensive item like a bicycle or cow is purchased?	2		4	5	1
Allowed to go alone to meet your friends for any reason?	3		3	2	
Are you permitted to visit women in other neighborhoods to talk with them?	4				7
Who in household decides to pay school fees for a relative from your side of family?	5	6		10	6
Who do you consult with for decisions regarding your children’s health care?	6	3		7	2
Allowed to buy things in the market without asking partner?	7			1	
In last 12 months, how often you and husband discussed children’s expenses	8	8		4	3
Can husband withdraw money from your bank account without consulting you?	9			9	
A wife should obey her husband, even if she disagrees.	10	4		3	

5-question standardized index corr. coeff.	.535	.4	.468	.5	.485
5-question model prediction index corr. coeff.	.538	.43	.49	.507	.5

**Table A.7:** Correlation of indices based on full sample versus data from one interviewer.

	Main results			Interviewer 1 only			Interviewer 2 only		
	LASSO stability selection	Random forest selection	Backward sequential selection	LASSO stability selection	Random forest selection	Backward sequential selection	LASSO stability selection	Random forest selection	Backward sequential selection
LASSO stability selection	1.000								
Random forest selection	0.942	1.000							
Backward sequential selection	0.832	0.841	1.000						
LASSO stability selection (Int. 1)	0.848	0.833	0.545	1.000					
Random forest selection (Int. 1)	0.839	0.883	0.605	0.882	1.000				
Backward sequential selection (Int. 1)	0.325	0.252	0.186	0.378	0.368	1.000			
LASSO stability selection (Int. 2)	0.770	0.818	0.880	0.517	0.644	0.192	1.000		
Random forest selection (Int. 2)	0.848	0.833	0.545	1.000	0.882	0.378	0.517	1.000	
Backward sequential selection (Int. 2)	0.751	0.798	0.880	0.477	0.569	0.113	0.966	0.477	1.000

**Table A.8:** Correlation of indices based on full sample versus data from one coder

	Main results			Coder 1 only			Coder 2 only		
	LASSO stability selection	Random forest selection	Backward sequential selection	LASSO stability selection	Random forest selection	Backward sequential selection	LASSO stability selection	Random forest selection	Backward sequential selection
LASSO stability selection	1.000								
Random forest selection	0.942	1.000							
Backward sequential selection	0.832	0.841	1.000						
LASSO stability selection (Coder 1)	0.953	0.964	0.805	1.000					
Random forest selection (Coder 1)	0.793	0.825	0.588	0.844	1.000				
Backward sequential selection (Coder 1)	0.835	0.808	0.682	0.826	0.697	1.000			
LASSO stability selection (Coder 2)	0.736	0.630	0.794	0.632	0.427	0.696	1.000		
Random forest selection (Coder 2)	0.788	0.822	0.809	0.808	0.610	0.646	0.693	1.000	
Backward sequential selection (Coder 2)	0.685	0.575	0.748	0.617	0.383	0.688	0.947	0.629	1.000

**Table A.9:** LASSO stability selection using binary scores within domains.

Question	Main results	Binary coding
Opinion heard when expensive item like a bicycle or cow is purchased?	1	5
Need permission from other household members to buy clothing for self?	2	3
Allowed to buy things in the market without asking partner?	3	1
Are you permitted to visit women in other neighborhoods to talk with them?	4	2
Who do you consult with for decisions regarding your children’s health care?	5	7
Are you permitted to visit any place riding on public transport?	6	4
Allowed to go alone to meet your friends for any reason?	7	8
Can decide by self to purchase emergency medicine for child	8	
Are you allowed to go alone to a relative’s house inside the village?	9	
When husband has different opinion, voice opinion and argue more often than voice opinion but do as he says	10	6

5-question standardized index corr. coeff.	.537	.515
5-question model prediction index corr. coeff.	.538	.518

*Notes*: Each domain is scored as a binary if the 4-value score is ⩾3.

**Table A.10:** Random forest selection using binary scores within domains.

Question	Main results	Binary coding
Are you permitted to visit any place riding on public transport?	1	1
Allowed to buy things in the market without asking partner?	2	2
Opinion heard when expensive item like a bicycle or cow is purchased?	3	3
Are you permitted to visit women in other neighborhoods to talk with them?	4	4
Need permission from other household members to buy clothing for self?	5	8
When was the last time you were unwell and visited a healthcare provider?	6	10
In last 12 months, how often you and husband discussed children’s expenses	7	5
Are you allowed to go alone to a relative’s house inside the village?	8	6
Allowed to go alone to meet your friends for any reason?	9	
Can decide by self to purchase emergency medicine for child	10	

5-question standardized index corr. coeff.	.501	.505

*Notes*: Each domain is scored as a binary if the 4-value score is ⩾3.

**Table A.11:** Backward sequential selection using binary scores within domains.

Question	Main results	Binary coding
Need permission from other household members to buy clothing for self?	1	9
Opinion heard when expensive item like a bicycle or cow is purchased?	2	4
Allowed to go alone to meet your friends for any reason?	3	8
Are you permitted to visit women in other neighborhoods to talk with them?	4	2
Who in household decides to pay school fees for a relative from your side of family?	5	
Who do you consult with for decisions regarding your children’s health care?	6	3
Allowed to buy things in the market without asking partner?	7	1
In last 12 months, how often you and husband discussed children’s expenses	8	5
Can husband withdraw money from your bank account without consulting you?	9	10
A wife should obey her husband, even if she disagrees.	10	6

5-question standardized index corr. coeff.	.535	.519
5-question model prediction index corr. coeff.	.538	.527

*Notes*: Each domain is scored as a binary if the 4-value score is ⩾3.

**Table A.12:** Selected survey questions using lab game.

Question	LASSO stability selection	Random forest selection (VSURF)	Backward sequential selection
	(1)	(2)	(3)
He (husband) expects me not to contradict him in public	1	3	1
Since New Year’s Day, how often has this (husband threatening to hurt you) happened?	2		2
On which step are you? (0 = powerless to 10 = most ability)	3	2	3
Are you allowed to go alone to a relative’s house inside the village?	4		4
Opinion heard when expensive item like a bicycle or cow is purchased?	5		
He is upset if I express an opinion that disagrees with him		1	
Often when disagree what to do, do what partner wants		4*	
Chose not to perform household roles in last 2 weeks		5*	
Has partner/other male ever threatened to hurt or harm you?			5

5-question standardized index corr. coeff.	0.213	0.106	0.070
5-question model prediction index corr. coeff.	0.218	0.680	0.219

*Notes*: The table lists the top 5 survey questions selected. The numbers in the cells indicate the selection order, with 1 referring to the best, or most predictive question. * indicates that the variable, when coded so that a higher value maps to more agency, has a negative correlation with the WTP measure. The reported correlation coefficients are between the agency measure from the lab game and the five-question index.

**Table B.1:** Full list of close-ended questions measuring agency.

**Question**	**Responses**
Who do you consult with for decisions regarding your children’s health care? *	${}^{1}$1. No one, I decide on my own 2. My husband. 3. Mother-in-law 4. Father-in-law 5. Relatives from my husband’s side -96. Other (specify): _______ -98. Don’t know -99. Refused to answer
Imagine that you were home alone, without your (*prefill response from previous question*) and one of your children was very sick. Could you make the choice *on your own* to purchase medication to treat your child?	∗1. Yes 2. No -98. Don’t know -99. Refused to answer
When was the last time you were unwell and visited a health care provider for treatment?	________ [DD] −98. Don’t know/ Can’t remember _________ [MM] −98. Don’t know/ Can’t remember _________ [YY] −98. Don’t know/ Can’t remember −99. Refused to answer
Who accompanied you to the provider? *Surveyor: Do not read out the response options aloud*	${}^{2}$ 1. Went alone 2. Husband 3. Mother-in-law 4. Sister-in-law 5. Father-in-law 6. Brother-in-law 7. Son 8. Father 9. Brother 10. Mother 11. Sister 12. Male relative 13. Female relative 14. Male non-relative 15. Female non-relative -98. Don’t know/ Can’t remember -99. Refused to answer
Getting permission to go?	1. Big problem 2. Small problem 3. No problem −98. Don’t know −99. Refused to answer
Finding someone to go with you?	1. Big problem 2. Small problem 3. No problem −98. Don’t know −99. Refused to answer
When you have small amounts of money, such as 20 or 50 INR, can you decide how to spend it on your own?	∗1. Yes, always 2. Yes, usually 3. Yes, sometimes 4. Rarely 5. Very rarely 6. Never -98. Don’t know −99. Refused to answer
When an expensive item like a bicycle or a cow is purchased by the household, is your opinion listened to in the decision of what to buy?	∗1. Yes, always 2. Yes, usually 3. Yes, sometimes 4. Rarely 5. Very rarely 6. Never -98. Don’t know −99. Refused to answer
If you have some money you have earned, can you use it to purchase clothing for yourself or children without asking the permission of anyone else?	∗1. Yes, always 2. Yes, usually 3. Yes, sometimes 4. Rarely 5. Very rarely 6. Never -98. Don’t know −99. Refused to answer
Are you allowed to buy things in the market without asking the permission of your partner?	∗1. Yes, always 2. Yes, usually 3. Yes, sometimes 4. Rarely 5. Very rarely 6. Never -98. Don’t know −99. Refused to answer
If money is available, who in your household decides whether to pay school fees for a relative from your side of the family?	∗1. You 2. You and your husband 3. You and someone other than husband 4. Husband 5. Husband with others -96. Other (specify): ______ -99. Refused to answer
If money is available, who in your household decides whether to purchase items like a radio or a paraffin lamp?	∗1. You 2. You and your husband 3. You and someone other than husband 4. Husband 5. Husband with others -96. Other (specify): ______ -99. Refused to answer
Do you have to ask the permission of other household members to buy: Vegetables or fruits	${}^{3}$ 1. Yes 2. No 3. Have never bought -99. Refused to answer
Do you have to ask the permission of other household members to buy: Clothing for yourself	1. Yes 2. No 3. Have never bought −99. Refused to answer
Do you have to ask the permission of other household members to buy: Medicines for yourself	1. Yes 2. No 3. Have never bought −99. Refused to answer
Do you have to ask the permission of other household members to buy: Personal supplies (soap, shampoo, dental paste, sanitary napkins, etc.)?	1. Yes 2. No 3. Have never bought −99. Refused to answer
Do you have access to any cash available now for buying HH food or medicine if you suddenly needed something?	∗1. Yes 2. No −98. Don’t know −99. Refused to answer
How much money do you usually have on hand to meet these types of expenses?	__________ -99. Refused to answer
Do you have a bank or savings account that you yourself use?	∗1. Yes 2. No −99. Refused to answer
Who makes deposits of money into this account?	∗1. You 2. You and your husband 3. Your husband −96. Other (specify): _____ -98. Don’t know -99. Refused to answer
Can your husband withdraw money from this account without consulting you?	1. Yes, always 2. Yes, usually 3. Yes, sometimes 4. Rarely 5. Very rarely 6. Never −98. Don’t know −99. Refused to answer
Can you withdraw money from this account without consulting your husband?	∗1. Yes, always 2. Yes, usually 3. Yes, sometimes 4. Rarely 5. Very rarely 6. Never -98. Don’t know -99. Refused to answer
Are you permitted to visit any place riding on public transport?	1. Never 2. Yes, but never alone 3. Yes, alone, with permission 4. Yes, alone, do not need permission -97. Not applicable −99. Refused to answer
Are you permitted to visit women in other neighborhoods to talk with them?	1. Never 2. Yes, but never alone 3. Yes, alone, with permission 4. Yes, alone, do not need permission -97. Not applicable -99. Refused to answer
Can you go unescorted to your parents’ house/village?	∗1. Yes 2. No -97. Not applicable -99. Refused to answer
Can you go unescorted to the next village?	∗1. Yes 2. No -99. Refused to answer
Are you allowed to go alone to a relative’s house inside the village?	∗1. Yes 2. No -97. Not applicable -99. Refused to answer
Are you allowed to go to the school/college alone or with friends?	∗1. Yes 2. No −97. Not applicable −99. Refused to answer
Are you allowed to go alone to meet your friends for any reason (to get school notes, chat, play etc.)?	∗1. Yes 2. No -97. Not applicable -99. Refused to answer
In the last one year, have you ever gone to the market within your village to buy personal items with friends? (no guardians)	1. Yes 2. No -97. Not applicable -99. Refused to answer
In the last one year, have you ever gone to the market within your village to buy personal items alone?	∗1. Yes 2. No -97. Not applicable -99. Refused to answer
In the last one year, have you ever attended any sort of community events/activities? (Ex: fair, theatre, cultural program, religious event)	∗1. Yes 2. No -97. Not applicable -99. Refused to answer
In the last one year, have you ever attended one of these events without guardians present (either alone or with friends)?	∗1. Yes 2. No -97. Not applicable -99. Refused to answer
A wife should obey her husband, even if she disagrees.	1. Strongly agree 2. Agree 3. Neither agree nor disagree 4. Disagree 5. Strongly disagree -98. Don’t know -99. Refused to answer
It is the job of men to be leaders, not women	1. Strongly agree 2. Agree 3. Neither agree nor disagree 4. Disagree 5. Strongly disagree -98. Don’t know -99. Refused to answer
A woman should be able to choose her own friends, even if her husband disapproves	∗1. Strongly agree 2. Agree 3. Neither agree nor disagree 4. Disagree 5. Strongly disagree -98. Don’t know -99. Refused to answer
In the last 12 months, approximately how often have you and your husband discussed […]? G.5. Children’s expenses G.6. Children’s education G.7. Your husband’s alcohol consumption G.8. Your husband’s relatives G.9. Your relatives G.11. Health expenses	1. Everyday 2. Once a week 3. Once a month 4. Every couple of months 5. Almost never 6. Never, we never talk about this subject 7. Never, we always agree about this subject -97. Not applicable -98. Don’t know -99. Refused to answer
Now I am going to read a list of things that might describe your current partner. Please tell me how closely this describes your current partner. G.12. Most of the time when we disagree about what to do, we do what my partner wants to do. G.13. My partner treats me well. G.14. My partner won’t let me wear certain things. G.15. When my partner and I are together, I’m pretty quiet. G.16. He expects me not to contradict him in public. G.17. He is upset if I express an opinion that disagrees with him. G.18. [Follow up to previous] I often express my opinion when I disagree with my husband. G.19. My partner has more say than I do about important decisions that affect us. G.20. My partner tells me who I can spend time with. G.21. I feel trapped or stuck in our relationship. G.22. My partner does what he wants, even if I do not want him to. G.23. When my partner and I disagree, he gets his way most of the time. G.24. My partner always wants to know where I am.	1. Strongly agree 2. Agree 3. Neither agree nor disagree/Neutral 4. Disagree 5. Strongly disagree -98. Don’t know −99. Refused to answer
When your husband has a different opinion from you on a particular decision what do you do. Please tell us how many times you adopt each of these approaches (*Surveyor: guide her through all the six options*) G.26. Don’t voice your opinion but do what you think is right G.27. Don’t voice your opinion and wait for another occasion to see if he changes his mind G.28. Don’t voice your opinion but do what your husband thinks is right G.29. Voice your opinion but also make it clear that you will go along with his view G.30. Voice your opinion and argue why your choice is better G.31. Other: Please explain	
	${}^{4}$1. Never 2. Rarely 3. Sometimes 4. Often 5. Most of the times 6. All the time -97. Not Applicable -98. Don’t know -99. Refused to answer
When you disagree with your husband, does he get angry with you?	1. Yes 2. No -99. Refused to answer
How often does he get angry with you when you disagree?	1. All the time 2. Almost all of the time 3. Some of the time 4. Once in a while 5. Rarely 6. Never -99. Refused to answer
Has he (your husband, or other adult male in your household) ever threatened to hurt or harm you or someone close to you?	1. Yes 2. No -99. Refused to answer
Since New Year’s Day, has this happened often, sometimes, rarely, or never?	1. Often 2. Sometimes 3. Rarely 4. Never -99. Refused to answer
It’s wrong for me to question people who are in charge or in authority, like teachers or parents, leaders in the village etc.	1. Strongly agree 2. Agree 3. Neither agree nor disagree 4. Disagree 5. Strongly disagree -98. Don’t know 99. Refused to answer
Do you feel that people like yourself can generally change things in your community if they want to?	∗1. Yes, very easily 2. Yes, fairly easily 3. Yes, but with a little difficulty 4. Yes, but with a great deal of difficulty 5. No, not at all -98. Don’t know −99. Refused to answer
Do you participate in making major household purchases? Major household purchases include things like refrigerator, television, vehicle, livestock etc.?	∗1. Yes 2. No -99. Refused to answer
I do not participate in making major household purchases…	∗1. Because I don’t like doing/ can’t/ don’t want to do it 2. Because it is not my responsibility 3. Because I don’t think it is important for me to make major household purchases 4. Because others don’t expect/ want me to make major household purchases 5. Because I will get in trouble if I do/ am not allowed to −96. Other (specify): _____ -98. Don’t know −99. Refused to answer
Thinking about your role in the household and the expectations of you, in the past 2 weeks have you ever *chosen for yourself* not to perform any of your roles or responsibilities?	1. Never 2. One or two times 3. Several times 4. Often 5. Nearly every day -98. Not sure/don’t know -99. Refused to answer
This is a ten step ladder, where on the bottom, the first step, stand people who are completely coerced or powerless, and on the highest step, the tenth step, stand those with the most ability to advance goals that they value in their own homes and in the world. On which step are you today?	Response Options: 1–10 -98. Don’t know −99. Refused to answer

*For this question as asked, lower-valued responses represent more agency. The response is then reverse-coded in the data analysis so that for all variables, a higher value represents more agency.

^1^Recoded as: 1. Relatives from my husband’s side, father-in-law, or mother-in-law, 2. My husband, 3. No one, I decide on my own.

^2^Recoded as: 1. With male relative, 2. With female relative, 3. Went alone.

^3^Recoded as: 1. With male relative, 2. With female relative, 3. Went alone.

^4^Recoded as four binary variables for yes/no questions: When husband has different opinion, voice opinion and argue more often than voice opinion but do as he says; When husband has different opinion, voice opinion but do as he says more often than not voicing opinion and waiting for him to change mind; When husband has different opinion, wait for him to change mind more often than do as he says (but don’t voice opinion); When husband has different opinion do what you think more often than what he says (but don’t voice opinion).

*Notes*: Table lists the 63 closed-ended questions used as the set from which the best 5 were selected.

## Appendix C Semi-structured interview guide

Thank you very much for speaking with me. My name is [NAME] and I work for an organization that works around the world to reduce poverty. The name of the organization is J-PAL. In India the organization’s office is in Delhi. We are not from the government.

**Purpose of the study:** The organization I work for conducts research all over India, and the results from the research are used to make better government programs. [Give example of immunization study in Haryana].

In this study, we are talking to many women in and around Kurukshetra. The reason we are doing this is to understand your experiences, your circumstances, your needs, and your struggles. We want to know how families make decisions on day-to-day events, such as child-rearing, household finances, health care issues in the family etc.

**What will you have to do?** We will talk to you for 30–45 min. We request that you answer as honestly as you can. Some of the questions might be of a personal or sensitive nature. If you ever feel uncomfortable, I want to remind you that it is okay to not answer. You should not feel obligated or pressured. We can stop the interview at any time. There will be no negative consequences if you stop the interview.

**Why are we recording?** We will also record the interview. It is difficult to talk and write notes at the same time. If I don’t record, I have to keep asking you what you said, and the interview will take longer. Because of the recorder, I can give you my full attention.

**What is being done to maintain confidentiality?** What you tell us, and the recording will only remain with our small research team. Maximum 3 or 4 people will be able to listen to the recording. Your name, your family members’ name, your address, the name of the village or anybody’s phone number, nothing will be in the recording. If I do something like that, I will be thrown out of my job. Our company takes the matter of confidentiality very seriously.

**Why do we need privacy?** I am requesting that we speak to you alone. There are two reasons for this. First, we have been told that we should only get the experiences of those mothers who have young children. That’s why we are talking only to you in your family. Second, if too many voices come into the recording, then it is difficult to listen to it later.

**Why are you only talking to me and not other village women?** We have selected you through a lottery. First, we selected about 20 villages in and around Kurukshetra through a lottery. Then we selected families within villages through a lottery.

**Any questions?** If you have any questions you may ask now or later. If you wish to contact later, please keep these numbers [give her the Contact Sheet].

### C.1 Introduction

*These questions are a way to start the conversation and are also important for the interviewer to remember for upcoming questions about the respondent’s work, children, and potential decision-makers within the household. Do not spend too much time on these questions as they are meant to be introductory and brief.*

*Note: The respondent may have already answered these questions in the quant survey. Say that she may already have answered these questions, and these are being asked again to refresh the researcher’s memory.*

• How old are you?
• How many years has it been since you got married?
• How many children do you have? How many boys and how many girls? How old are they?
• Besides yourself, your husband, and children, how many people live in your household? Can you tell me who they are?
• Does your husband work? If yes, what work?
• Do you work outside the home? If yes, what work? *[Throughout interview, probe about working where appropriate.]*

### C.2 Children’s Schooling

*The questions in this section are to understand the extent of involvement of the mother in decisions related to her children’s schooling.*

*The goal is to engage in a conversation to ascertain how engaged the mother is in these decisions, how she negotiates, the extent to which she cedes control, to whom she cedes control, whether she thinks of it as ceding control, etc. The ‘facts’ that are embedded in the question – whether it is a private or government school or how much it costs (which can be answered in a survey as well) – are conversation starters to get deeper into questions about decision-making power. The sub-questions are meant to ask for more details that should lead to a fuller account, a narrative, or a story. If they do not work, you can modify. Do not ask them like they are questions in a survey.*

*The questions will be asked separately for sons and daughters to understand gender differences. Note that we are only asking about her children under the age of 10. If there is no school age child, skip this section.*

The next few questions are about your children – their schooling and healthcare. First, I’ll ask about your daughter’s education.

• Which school does she go to? Is it a government or private school? Does it cost money to go to this school? How much?
• Who decided on this school?
• Why did you choose this school?
• Did you think about any other schools? Which ones? *[If there were other schools in consideration, probe about how choices were made, and why.]*
• Was there agreement among family members about the school? If not, ask for details about the actors and events around the disagreement.
• Did you agree with the decision? Why/why not?
• Do you ever have to keep your daughter from going to school? I am not asking about when she is sick…for any other reason?
• Till what class do you want your daughter to study? Why?
• What are your hopes and aspirations for your daughter? Do you think you can help her fulfill these aspirations? Why/why not?

*If you do not get much traction with the schooling questions, ask about aspirations for the daughter post-puberty.*

Let’s now talk about your son’s education.

• Which school does he go to? Is it a government or private school? Does it cost money to go to this school? How much?
• Who decided on this school?
• Why did you choose this school?
• Did you think about any other schools? Which ones? *[If there were other schools in consideration, probe about how choices were made, and why.]*
• Was there agreement among family members about the school? If not, ask for details about the actors and events around the disagreement.
• Did you agree with the decision? Why/why not?
• Do you ever have to keep your son from going to school? I am not asking about when he is sick…for any other reason?
• Till what class do you want your son to study? Why?
• What are your hopes and aspirations for your son? Do you think you can help him fulfill these aspirations? Why/why not?

### C.3 Children’s health care

*Similar to Section 2, this section is also about the extent of involvement of the mother in decisions related to her children, in this case, the child’s health care.*

*Again, the goal is to engage in a conversation and get a narrative account of an actual incident that involved her child. Any specific details that are “factual” – type of illness, which doctor, who took the child to the doctor etc. – are the bridge to allow the woman to talk in depth about her own engagement with all the small decisions that are involved in getting to the big picture of the decision-making process, her own control over these decisions, whether she cedes control, how much, and her opinions on the same.*

*Questions on the respondent’s mobility are embedded within the questions and should be probed.*

*The questions will be asked separately for sons and daughters to understand gender differences.*

I will now ask about your children’s health care.

• Can you remember the last time your child was sick, and you had to take your child to the doctor? Can you tell me about what happened?
  -Was it your son or daughter? When did this happen? How old was your child? What was the sickness? Which doctor or clinic or hospital did you take your child to?
  -Who decided on which doctor/hospital? Why?
  -Were you in agreement with the decision? Why?
  -Who took the child to the doctor? Why?
  -If she did not go, ask whether she wanted to go. If she wanted to go, ask why she did not go. If she did not want to go, ask why she did not want to go.
  -What was the treatment? Who took care of the treating the child?
  -Were you satisfied with the treatment? Why/why not?
• If the woman has a child of another gender, ask the same questions about that child.

*If she has an infant, you can ask about vaccination.*

### C.4 Fertility

*The questions should probe about respondent’s choice and agency around the number of children, birth spacing, and decisions around breastfeeding.*

• You said you have X children. Would you like to have more? Why/why not? Would your husband (in-laws; whoever she says is in charge) agree with your decision? If not, why not?
• According to you, what is the ideal spacing between children? *[If there is a discrepancy between what she says is the ideal and we know to be the fact, probe about the discrepancy.]* Whose decision was it to have a different spacing?
• Ask about contraception.
• Ask separately for son and daughter. Did you breastfeed daughter/son? For how long? Probe about decisions related to breastfeeding – who decided how long to breastfeed? Did she agree with the decision? Were there any disagreements in the household about this decision? Why? If she did not breastfeed, ask why.
• Ask about delivery – at home or institutional?

### C.5 Household expenses

*These questions are around a woman’s control (or lack thereof) over household budgets, and her involvement in decisions around making purchases of various sorts.*

*There’s a separation between purchasing smaller and larger items for the household. Determine what the appropriate small or large item is likely to be for the household and probe accordingly.*

*Questions on the respondent’s mobility are embedded within the questions and should be probed.*

Finally, I’d like to ask about household expenses.

• Overall budgeting questions
  -How do you run the household?
  -If she works, asks if she hands over her pay or keeps it? Who is in charge of the household money?
• Buying items of daily need
  -Who is in charge of the money for buying items needed on a daily or regular basis?
  -Wheat, vegetables, milk, soap etc. Who goes out to buy these items?
  -If she goes, would she prefer that somebody else was in charge of doing the shopping?
  -If she doesn’t go, would she like to go? If she would like to go, why is she not able to?
  -Does she have a say in what items get purchased?
  -After marriage, did she have a say if she needed anything that she was used to buying before?
  -Any particular vegetable she liked, or any brand of soap?
• Buying items in an emergency
  -Questions about the different ways that the woman saves (buffalo milk money is hers, separate bank account for girl child through a government scheme, gullak etc.) are yielding responses, so continue asking about this.
• Buying a large item for the household
• What if the household won a lottery?

*Be attuned to any issue she raises about running debt with the local shop or money lenders and probe about how this plays into decision-making in household budgeting decisions.*

### C.6 Mobility

*The goal of adding this section on mobility is to understand the constraints placed on women’s physical mobility, and whether, and to what extent, she has agency in her own movement.*

• When you go to visit your family/natal village, how do you go (means of transport)? Do you go by yourself or does someone need to accompany you? Probe about why.
• *[DRAW A MOBILITY MAP.]*

These are all the questions. Do you have any questions for me? Thanks very much for participating in this interview.

## Footnotes

1 We follow the literature on how to conceptualize women’s agency, focusing on her agency within her household and marriage (Rowlands, 1997, Kabeer, 1999). The next section reviews the literature that conceptualizes agency.

2 The more common ways to assess measures draw on content validity and construct validity. With content validity, experts use their subjective judgment to assess whether a measure covers all facets of the construct. With construct validity, the researcher uses theory to predict that a construct is related to another variable. One might posit that women’s agency is related to another factor *Z* because *Z* increases agency or agency increases *Z*. Then one judges a measure of agency based on its correlation with *Z*. The advantage of this approach is the researcher almost always has data for some candidate *Z*. The disadvantage is we are rarely certain that women’s agency causes or is caused by *Z*. For example, it is often hypothesized that education confers more agency, but it is theoretically possible that this link is weak.

3 Five questions seems to align with the length many survey designers seek for a short module, but the method is flexible to increase or decrease this length.

4 *Masi* means maternal aunt in Hindi.

5 McBride and Nichols (2018) also use machine learning to design a survey-based proxy for poverty, and Knippenberg, Jensen, and Constas, 2019 do so for food insecurity.

6 See also Camfield et al., 2009, Small et al., 2008, Kanbur and Shaffer, 2007, Rao, 2002, Shaffer, 2013. Researchers might collect qualitative data from the study population as a first step and use it to design new survey questions or improve the validity of proposed questions through open-ended debriefing techniques during piloting of questions. Techniques include interviews and group discussions with respondents about how they understood the questions, asking them to think aloud as they answer them, or having a panel of experts review the questions (Bowden et al., 2002, Durham et al., 2011, Latcheva, 2011, Cohen and Saisana, 2014, Greco et al., 2018).

7 Using the 2011 Census, of the 407 villages in Kurukshetra district, we excluded the top and bottom 5% of villages based on population, distance to the district headquarters, child sex ratio, and female literacy rate. We also excluded a few villages with similar names as to avoid confusion in the field. Among the remaining 303 villages, we picked 2 or 3 villages in each of 8 strata, defined by being above or below median population, distance to district headquarters, and ratio of male to female literacy.

8 A few women declined to participate in the second part of the data collection or the second team could not locate them. The sample of 209 qualitative interviews are those for whom we also have quantitative data. We conducted qualitative interviews with 9 additional women for whom the quantitative data are missing.

9 If more than one woman in a household was eligible, we randomly selected one to participate in the study.

10 Some questions have missing responses, primarily due to skip patterns in the survey. Missing data due to “don’t know” or “refuse to answer” is uncommon. For one variable, that asks the date of the last medical appointment, 10 of the 209 women have a “don’t know” missing value. Of the remaining 62 variables, 52 variables have no missing observations due to “don’t know” or “refuse to answer” responses, 2 variables have 2 missing observations, and 8 variables have 1 missing observation. To include these questions in our analysis, we impute the value with the sample mean. To inform this decision, we estimated a standard LASSO model that included missing flags among the potential predictors. Because few of them were selected as predictors, we did not include them in our main analyses.

11 When we developed the coding approach by triply coding ten transcripts, we tried using a scale of 1 to 3, 1 to 4, and 1 to 5. We chose 1 to 4 because it seemed to best capture the nuances in the interviews and to allow us to define each rank distinctly.

12 We test robustness to creating a standardized index across the six domains in Section 6.

13 We ask about amounts for her husband between ₹ 100 and ₹ 700. We inform her that any transfer of money to her will take place privately and that we will not communicate with her husband about the game if she chooses money for herself. If she chooses for her husband to get the money, we will give it to him and explain that it is tied to his wife’s participation in our study.

14 Supervised machine learning uses labeled data to train the model. The qualitative scores serve as the labels in our analysis. Another approach would be to use only the quantitative survey questions as data and apply unsupervised machine learning techniques for feature selection (Solorio-Fernández, Carrasco-Ochoa, & Martínez-Trinidad, 2020).

15 As a brief primer on LASSO, it is a type of regularized regression. A regularized regression differs from a standard regression in that the estimator “shrinks” some coefficients toward zero to avoid the model over-fitting the data. LASSO shrinks some coefficients all the way to zero; starting from a large set of regressors, only a subset will have non-zero coefficient estimates, or are selected for inclusion in the model. The tuning parameter specifies how aggressive the procedure should be in shrinking coefficients.

16 Implemented in Stata on a standard desktop computer, the procedure takes 19 min to run. Backward sequential selection takes a few seconds. Random forest selection, implemented in R, takes 15 min.

18 With random forest, one builds decision trees to classify or fit the data. At each node of a tree, one of the variables is used to partition the data. Only a random subset of potential variables is considered at each split, and the one that best partitions the data is used. A random forest is an ensemble of many trees. For each tree, some observations are left out, and the predictions are validated against this “out of bag” sample.

19 In addition to this performance-based approach to using random forest for feature selection, there are approaches that use only variable importance, such as the one proposed by Strobl, Boulesteix, Kneib, Augustin, and Zeileis (2008).

24 In our application, 42 of the full set of 63 variables are retained at the end of step 1, and 13 of those variables are retained at the end of step 2.

25 One can also run sequential selection in the forward direction, starting with an empty set and then sequentially adding the most predictive variable among the candidates. Backward selection typically outperforms forward selection (Leslie, Zhou, Spiegelman, & Kruk, 2018).

26 We calculated the qualitative score by averaging the six domain-specific scores. We repeated the analysis using an alternative qualitative score that is a standardized index across the domains. This change did not alter the top five questions selected by any of the three algorithms. While this amount of insensitivity need not always hold, this result provides some additional reassurance about the robustness of our method.

27 The formula for combining our five questions is 1.02+0.071q1+0.200q2+0.049q3+0.117q4+0.167q5 where *qn* is the $n^{\mathrm{th}}$-ranked question.

28 The two new questions in the top five set for random forest are ranked sixth and twelfth by LASSO stability selection. The two new questions in the top five for backward sequential selection are ranked seventh and twelfth by LASSO stability selection.

29 R code that allows one to generate the predicted value from a random forest or LASSO model trained on our data is available from the corresponding author.

30 As an additional way to test sensitivity to subjectivity in the coding, we also repeated the analysis using a binary coding of each domain of the semi-structured interview, instead of a four-point scale. Appendix Table A.9, Table A.10, Table A.11 show that the results are very stable. For example, with LASSO stability selection, four of the top five questions overlap with the main analysis, and the correlation between this variant and the main index is 0.97.

31 The premise of the game is that -WTP is a measure of agency; the higher a woman’s WTP, the lower her agency. As a partial fix for the problem of some low-agency women not wanting to receive the money, we bottom code WTP at 0: the measure of agency is min{-WTP,0}. We also tried constructing a measure of agency in which either high or low WTP mapped to low agency, but the algorithm-selected survey indices were again low-performing. In addition, the lab game measure, either our main one or the non-monotonic alternative, have a low correlation with the qualitative score.

## Footnotes

17 The LASSO tuning parameter is chosen within each iteration by 5-fold cross-validation.

20 We use the default variable importance in the VSURF package written in R by Genuer, Poggi, and Tuleau-Malot (2015). It is the difference in out-of-bag error between trees built with the variable and those trees with the variable randomly permuted across observations, averaged across all trees in the forest that used the variable.

21 Variables with low average VI generally have a low standard deviation; the standard deviation rule is a more robust way to eliminate variables with low importance than doing so based on average VI. The threshold is calculated by estimating a decision tree (specifically CART) with 63 observations mapping to the available predictors. The dependent variable is the standard deviation of its VI, and the independent variable is its rank. The threshold is the minimum standard deviation predicted by the CART. Variables with a standard deviation below this threshold are eliminated.

22 The threshold is the sum of the minimum OOB error among the step 2 models (that vary in the number of included predictors) and the standard deviation of that model’s OOB error across the 100 forests.

23 The threshold is proportional to the change in OOB error between the model at the end of step 1 and the model at the end of step 2. The threshold also depends on a multiplicative tuning parameter.

## References

[b0005] Alkire S., Meinzen-Dick R., Peterman A., Quisumbing A., Seymour G., Vaz A. (2013). The women’s empowerment in agriculture index. World Development.

[b0010] Almås I., Armand A., Attanasio O., Carneiro P. (2018). Measuring and changing control: Women’s empowerment and targeted transfers. Economic Journal.

[b0015] Almås, I., Berge, L.I., Bjorvatn, K., Somville, V., and Tungodden, B. (2020). Adverse selection into competition: Evidence from a large-scale field experiment in tanzania. Discussion Paper Series in Economics 19/2020, Norwegian School of Economics, Department of Economics.

[b0020] Barr, A., Dekker, M., Mwansa, F., and Zuze, T.L. (2020). Financial decision-making, gender and social norms in Zambia: Preliminary report on the quantitative data generation, analysis and results. CeDEx Discussion Paper Series.

[b0025] Becker G.M., DeGroot M.H., Marschak J. (1964). Measuring utility by a single-response sequential method. Behavioral Science.

[b0030] Bhan, N., Thomas, E., Dixit, A., Averbach, S., Dey, A., Rao, N., Lundgren, R., Silverman, J., and Raj, A. (2020). Measuring women’s agency and gender norms in family planning: What do we know and where do we go?.

[b0035] Bowden A., Fox-Rushby J., Nyandieka L., Wanjau J. (2002). Methods for pre-testing and piloting survey questions: Illustrations from the KENQOL survey of health-related quality of life. Health Policy and Planning.

[b0040] Camfield L., Crivello G., Woodhead M. (2009). Wellbeing research in developing countries: Reviewing the role of qualitative methods. Social Indicators Research.

[b0045] Campbell C., Mannell J. (2016). Conceptualising the agency of highly marginalised women: Intimate partner violence in extreme settings. Global Public Health.

[b0050] Center on Gender Health and Equity (2020). A roadmap for measuring agency and social norms in women’s economic empowerment.

[b0055] Chambers R. (1994). Participatory rural appraisal (pra): Analysis of experience. World development.

[b0060] Cohen A., Saisana M. (2014). Quantifying the qualitative: Eliciting expert input to develop the Multidimensional Poverty Assessment Tool. Journal of Development Studies.

[b0065] Creswell, J.W. and Clark, V.L.P. (2017). Designing and conducting mixed methods research. Sage Publications, Los Angeles, 3 edition.

[b0070] Cronbach L.J., Meehl P.E. (1955). Construct validity in psychological tests. Psychological Bulletin.

[b0075] Deterding N.M., Waters M.C. (2018). Flexible coding of in-depth interviews: A twenty-first-century approach. Sociological Methods and Research.

[b0080] DeVon H.A., Block M.E., Moyle-Wright P., Ernst D.M., Hayden S.J., Lazzara D.J., Savoy S.M., Kostas-Polston E. (2007). A psychometric toolbox for testing validity and reliability. Journal of Nursing Scholarship.

[b0085] Donald A., Koolwal G., Annan J., Falb K., Goldstein M. (2020). Measuring women’s agency. Feminist Economics.

[b0090] Durham J., Tan B.-K., White R. (2011). Utilizing mixed research methods to develop a quantitative assessment tool: An example from explosive remnants of a war clearance program. Journal of Mixed Methods Research.

[b0095] Ewerling F., Lynch J.W., Victora C.G., van Eerdewijk A., Tyszler M., Barros A.J. (2017). The SWPER index for women’s empowerment in Africa: Development and validation of an index based on survey data. The Lancet Global Health.

[b0100] Ewerling F., Raj A., Victora C.G., Hellwig F., Coll C.V., Barros A.J. (2020). SWPER Global: A survey-based women’s empowerment index expanded from Africa to all low- and middle-income countries. Journal of Global Health.

[b0105] Genuer R., Poggi J.-M., Tuleau-Malot C. (2010). Variable selection using random forests. Pattern Recognition Letters.

[b0110] Genuer R., Poggi J.-M., Tuleau-Malot C. (2015). VSURF: An R package for variable selection using random forests. The R Journal.

[b0115] Glennerster, R., Walsh, C., and Diaz-Martin, L. (2018). A practical guide to measuring women’s and girls’ empowerment in impact evaluations. Technical report. Abdul Latif Jameel Poverty Action Lab.

[b0120] Gonzalez O. (2020). Psychometric and machine learning approaches for diagnostic assessment and tests of individual classification. Psychological Methods.

[b0125] Greco G., Skordis-Worrall J., Mills A. (2018). Development, validity, and reliability of the women’s capabilities index. Journal of Human Development and Capabilities.

[b0130] Guyon I., Weston J., Barnhill S., Vapnik V. (2002). Gene selection for cancer classification using support vector machines. Machine Learning.

[b0135] Jackson C. (2011). Research with experimental games: Questioning practice and interpretation. Progress in Development Studies.

[b0140] Jayachandran S. (2015). The roots of gender inequality in developing countries. Annual Review of Economics.

[b0145] Jose R., Bhan N., Raj A. (2017). Center on Gender Equity and Health (GEH).

[b0150] Kabeer N. (1999). Resources, agency, achievements: Reflections on the measurement of women’s empowerment. Development and Change.

[b0155] Kanbur R., Shaffer P. (2007). Experience of combining qualitative and quantitative approaches in poverty analysis. World Development.

[b0160] Kishor S., Gupta K. (2004). Women’s empowerment in India and its states: Evidence from the NFHS. Economic and Political Weekly.

[b0165] Knippenberg E., Jensen N., Constas M. (2019). Quantifying household resilience with high frequency data: Temporal dynamics and methodological options. World Development.

[b0170] Kshirsagar, V., Wieczorek, J., Ramanathan, S., and Wells, R. (2017). Household poverty classification in data-scarce environments: A machine learning approach. 31st Conference on Neural Information Processing Systems (NIPS 2017).

[b0175] Laszlo S., Grantham K., Oskay E., Zhang T. (2020). Grappling with the challenges of measuring women’s economic empowerment in intrahousehold settings. World Development.

[b0180] Latcheva R. (2011). Cognitive interviewing and factor-analytic techniques: A mixed method approach to validity of survey items measuring national identity. Quality & Quantity.

[b0185] Leslie H.H., Zhou X., Spiegelman D., Kruk M.E. (2018). Health system measurement: Harnessing machine learning to advance global health. PLoS ONE.

[b0190] Liu H., Motoda H. (1998).

[b0195] Madhok S. (2014).

[b0200] Maiorano D., Shrimankar D., Thapar-Björkert S., Blomkvist H. (2021). Measuring empowerment: Choices, values and norms. World Development.

[b0205] Malapit H., Quisumbing A., Meinzen-Dick R., Seymour G., Martinez E.M., Heckert J., Rubin D., Vaz A., Yount K.M., Phase G.A.A.P. (2019). Development of the project-level Women’s Empowerment in Agriculture Index (pro-WEAI). World Development.

[b0210] McBride L., Nichols A. (2018). Retooling poverty targeting using out-of-sample validation and machine learning. The World Bank Economic Review.

[b0215] Meinshausen N., Bühlmann P. (2010). Stability selection. Journal of the Royal Statistical Society: Series B (Statistical Methodology).

[b0220] Naybor D., Poon J.P., Casas I. (2016). Mobility disadvantage and livelihood opportunities of marginalized widowed women in rural Uganda. Annals of the American Association of Geographers.

[b0225] Nussbaum M. (1999). Women and equality: The capabilities approach. International Labor Review.

[b0230] Onwuegbuzie A.J., Bustamante R.M., Nelson J.A. (2010). Mixed research as a tool for developing quantitative instruments. Journal of Mixed Methods Research.

[b0235] Peterman A., Schwab B., Roy S., Hidrobo M., Gilligan D.O. (2021). Measuring women’s decisionmaking: Indicator choice and survey design experiments from cash and food transfer evaluations in Ecuador. Uganda and Yemen. World Development.

[b0240] Pulerwitz J., Gortmaker S.L., DeJong W. (2000). Measuring sexual relationship power in HIV/STD research. Sex Roles.

[b0245] Rahman L., Rao V. (2004). The determinants of gender equity in India: examining Dyson and Moore’s thesis with new data. Population and Development Review.

[b0250] Rao V. (2002). Experiments in ‘participatory econometrics’: Improving the connection between economic analysis and the real world. Economic and Political Weekly.

[b0255] Richardson R., Schmitz N., Harper S., Nandi A. (2019). Development of a tool to measure women’s agency in India. Journal of Human Development and Capabilities.

[b0260] Rowlands J. (1997).

[b0265] Ryan R.M., Deci E.L. (2000). Self-determination theory and the facilitation of intrinsic motivation, social development, and well-being. American Psychologist.

[b0270] Shaffer P. (2013). Ten years of ‘Q-Squared’: Implications for understanding and explaining poverty. World Development.

[b0275] Small M.L., Jacobs E.M., Massengill R.P. (2008). Why organizational ties matter for neighborhood effects: Resource access through childcare centers. Social Forces.

[b0280] Solorio-Fernández S., Carrasco-Ochoa J.A., Martínez-Trinidad J.F. (2020). A review of unsupervised feature selection methods. Artificial Intelligence Review.

[b0285] Strobl C., Boulesteix A.-L., Kneib T., Augustin T., Zeileis A. (2008). Conditional variable importance for random forests. BMC Bioinformatics.

[b0290] Vaz A., Pratley P., Alkire S. (2016). Measuring women’s autonomy in Chad using the relative autonomy index. Feminist Economics.

[b0295] Zhou Y. (2019). A mixed methods model of scale development and validation analysis. Measurement: Interdisciplinary Research and Perspectives.

